# Phylogenomic and Molecular Demarcation of the Core Members of the Polyphyletic *Pasteurellaceae* Genera *Actinobacillus*, *Haemophilus*, and *Pasteurella*

**DOI:** 10.1155/2015/198560

**Published:** 2015-03-03

**Authors:** Sohail Naushad, Mobolaji Adeolu, Nisha Goel, Bijendra Khadka, Aqeel Al-Dahwi, Radhey S. Gupta

**Affiliations:** Department of Biochemistry and Biomedical Sciences, McMaster University, Hamilton, ON, Canada L8N 3Z5

## Abstract

The genera* Actinobacillus, Haemophilus, *and* Pasteurella* exhibit extensive polyphyletic branching in phylogenetic trees and do not represent coherent clusters of species. In this study, we have utilized molecular signatures identified through comparative genomic analyses in conjunction with genome based and multilocus sequence based phylogenetic analyses to clarify the phylogenetic and taxonomic boundary of these genera. We have identified large clusters of* Actinobacillus, Haemophilus, *and* Pasteurella* species which represent the “*sensu stricto*” members of these genera. We have identified 3, 7, and 6 conserved signature indels (CSIs), which are specifically shared by* sensu stricto* members of* Actinobacillus, Haemophilus, *and* Pasteurella*, respectively. We have also identified two different sets of CSIs that are unique characteristics of the pathogen containing genera* Aggregatibacter* and* Mannheimia*, respectively. It is now possible to demarcate the genera* Actinobacillus sensu stricto, Haemophilus sensu stricto, *and* Pasteurella sensu stricto* on the basis of discrete molecular signatures. The other members of the genera* Actinobacillus, Haemophilus, *and* Pasteurella* that do not fall within the “*sensu stricto*” clades and do not contain these molecular signatures should be reclassified as other genera. The CSIs identified here also provide useful diagnostic targets for the identification of current and novel members of the indicated genera.

## 1. Introduction 

The family* Pasteurellaceae*, the single constituent family of the order* Pasteurellales*, represents a diverse group of commensal and pathogenic bacteria within the class* Gammaproteobacteria*. The family currently contains 19 genera, some of which are particularly important human and animal pathogens [1, 2]. The genera* Haemophilus* contains species responsible for human bacteremia, pneumonia, acute bacterial meningitis, and the sexually transmitted disease chancroid [3–5];* Aggregatibacter* species have been implicated in juvenile periodontitis [6]; members of the genera* Mannheimia*,* Pasteurella*, and* Actinobacillus* have been implicated in the causation of shipping fever in cattle, fowl cholera, and pleuropneumonia in pigs, respectively [7–9].

The family* Pasteurellaceae* was originally proposed as a higher level taxonomic grouping of the related pathogenic genera* Actinobacillus, Haemophilus, *and* Pasteurella *[10]. Classification of organisms into these three genera was primarily based on DNA G-C content and a handful of phenotypic traits [11]. The phenotypic traits were later found not to be characteristic of any single genus [12]. Consequently, the genera* Actinobacillus, Haemophilus, *and* Pasteurella* each exhibit extensive polyphyly in subsequent 16S rRNA based phylogenies [12, 13]. Additional studies based on individual or concatenated gene sets and DNA-DNA/rRNA-DNA hybridization also support the presence of extensive polyphyly within the genera* Actinobacillus, Haemophilus, *and* Pasteurella* [14–20].

Extensive work has been undertaken to amend the classification of the genera* Actinobacillus, Haemophilus, *and* Pasteurella *[1, 5, 9, 18]. New genera have been created to house phylogenetically coherent clusters of* Actinobacillus, Haemophilus, *and* Pasteurella*. The species [*Actinobacillus*]* actinomycetemcomitans*, [*Haemophilus*]* aphrophilus*, [*Haemophilus*]* paraphrophilus,* and [*Haemophilus*]* segnis* have been transferred to the genus* Aggregatibacter* [21]; the species [*Haemophilus*]* paragallinarum*, [*Pasteurella*]* gallinarum*, [*Pasteurella*]* avium,* and [*Pasteurella*]* volantium* have been transferred to the genus* Avibacterium *[22]; the species [*Haemophilus*]* somnus* and [*Haemophilus*]* agni* have been transferred to the genus* Histophilus *[23]; and the species [*Pasteurella*]* haemolytica* and [*Pasteurella*]* granulomatis *have been transferred to the genus* Mannheimia *[7]. Additionally, some individual species within the genera* Actinobacillus, Haemophilus, *and* Pasteurella* that do not cluster with other members of their genus in phylogenetic trees have been moved or proposed to be moved to novel or neighbouring genera (namely, the transfer of the species [*Haemophilus*]* pleuropneumoniae* to the genus* Actinobacillus *[24], the transfer of the species [*Pasteurella*]* anatis* to the genus* Gallibacterium *[25], the transfer of the species [*Pasteurella*]* trehalosi *to the genus* Bibersteinia *[26], the transfer of the species [*Pasteurella*]* ureae *to the genus* Actinobacillus* [27], and the proposed transfer of the species [*Haemophilus*]* ducreyi* to a novel genus [28]). However, despite these changes, the classification of the genera* Actinobacillus, Haemophilus, *and* Pasteurella* is still problematic and each genus continues to contain members which exhibit polyphyletic branching [5, 17–20].

Multiple studies have attempted to define a core group of species which cluster around the nomenclatural type species of* Actinobacillus, Haemophilus, *or* Pasteurella* as the only true members of these genera (i.e.,* sensu stricto*) [13, 15–17, 29–31], but the taxonomy and phylogeny of these bacteria continue to remain inconclusive [20, 32, 33]. Several methods have been employed for the demarcation of these genera; however, no simple method or criterion is available that can clearly delimit these genera. It has been suggested that genome based studies may provide reliable means of clarifying the evolutionary relationships of these bacteria [33].

Since the availability of the first complete genome sequence of the* Haemophilus influenzae* [34], a large number of genomes for the members of the family* Pasteurellaceae* have become available in public databases [35, 36]. The availability of these genomes provides us with an opportunity to complete comprehensive genome scale phylogenetic analyses of the family* Pasteurellaceae*. These genome sequences have also been utilized to carry out comparative genomic analyses to identify molecular signatures (namely, conserved signature indels (CSIs) in various proteins), commonly shared by all or closely related subsets of species within the family* Pasteurellaceae*. On the basis of the molecular signatures identified from comparative analyses of* Pasteurellaceae* genomes in conjunction with core genome based and multilocus sequence based phylogenetic analyses, we have identified* sensu stricto* clades of* Actinobacillus, Haemophilus, *and* Pasteurella* that are supported by 3, 7, and 6 unique molecular signatures, respectively. We also report sets of molecular signatures that are unique characteristics of the pathogen containing genera* Aggregatibacter* and* Mannheimia*.

## 2. Methods

### 2.1. Multilocus Sequence Analysis

Multilocus sequence analysis was completed for members of the family* Pasteurellaceae* using widely available nucleotide sequences of the 16S rDNA,* infB* (translation initiation factor IF-2),* recN* (DNA repair protein), and* rpoB* (DNA-directed RNA polymerase subunit beta) genes which have been used, individually or as part of a set, in a number of previous phylogenetic analyses of the family* Pasteurellaceae* [15–17, 29, 30]. Gene sequences for these four genes were obtained for 52* Pasteurellaceae* strains, representing a large majority of the known* Pasteurellaceae* species, and 2 members of* Vibrio cholerae* from the NCBI nucleotide database [37]. Species which were missing one of these four genes or which did not have a gene sequence that was at least 50% of the length of the full gene were excluded from the analysis. The four genes were individually aligned using MUSCLE [38] and manually concatenated to create a combined dataset that contained 10 183 nucleotide long alignments. A maximum-likelihood tree based on 100 bootstrap replicates of this alignment was constructed using MEGA 6.0 [39] while employing maximum composite likelihood substitution model.

### 2.2. *Pasteurellaceae* Core Genome Phylogenetic Tree

A phylogenetic tree of 76* Pasteurellaceae* strains, rooted using 7 members of the family* Vibrionaceae*, based on the core genome of the family* Pasteurellaceae* was created for this study. The core set of* Pasteurellaceae* proteins were identified using the UCLUST algorithm [55] to identify widely distributed protein families with at least 30% sequence identity and 50% sequence length. Proteins families which were present in less than 50% of the input genomes were excluded from further analysis. Potentially paralogous sequences (additional proteins from the same organism in a single protein family) within the remaining protein families were also excluded from further analysis. Each protein family was individually aligned using MAFFT 7 [56]. Aligned amino acid positions which contained gaps in more than 50% of organisms were excluded from further analysis. The remaining amino acid positions were concatenated to create a combined dataset that contained 128 080 amino acid long alignments. An approximately maximum-likelihood tree based on this alignment was constructed using FastTree 2 [57] while employing the Whelan and Goldman substitution model [58].

### 2.3. Identification of Molecular Signatures (CSIs) for Different Genera of the Family* Pasteurellaceae*

The detailed outline of the process of identifying CSIs has been recently published [59]. In brief, Blastp searches were performed on all proteins from the genome of* Haemophilus influenzae *F3047 [47]. Ten to fifteen high scoring homologues that were present in* Haemophilus, *other* Pasteurellaceae*, and* Gammaproteobacteria* species were retrieved, and their multiple sequence alignments were constructed using Clustal X 1.83 [60]. The alignments were visually inspected to identify any conserved inserts or deletions (indels) that are restricted to the particular clades of the family Pasteurellaceae, which are flanked on each side by at least 5-6 identical/conserved residues in the neighbouring 30–40 amino acids. The selected sequences containing the indels and their flanking conserved regions were further evaluated by detailed Blastp searches to determine species distribution and group specificity. The results of these Blast searches were processed using Sig_Create and Seq_Style to construct signature files [59]. Due to space constraints, the sequence alignment files presented here contain sequence information for a limited number of species within the order* Pasteurellaceae *and a representative selection of outgroup species. However, in each case, all members of the order and outgroups exhibited similar sequence characteristics to the representatives.

## 3. Results and Discussion

### 3.1. Phylogenetic Analysis of the* Pasteurellaceae*

Elucidating an accurate phylogeny of the members of the family* Pasteurellaceae* has been a long standing challenge in* Pasteurellaceae* research [10–12, 18, 19]. Early 16S rRNA based studies revealed that the established taxonomy of the family* Pasteurellaceae* was not consistent with their genetically inferred phylogeny [12, 14]. This has led to a long series of taxonomic revisions within the family* Pasteurellaceae*, a process which is still taking place today [7, 18, 22, 28]. However, it was subsequently discovered that phylogenetic trees of* Pasteurellaceae* species based on different genes did not completely agree with each other [15, 16, 31]. In particular, phylogenetic trees based on the 16S rRNA gene, often considered the gold standard in bacterial taxonomy and phylogeny [61, 62], disagreed with highly robust multilocus sequence and concatenated protein sequence based phylogenetic trees [9, 17, 19, 20, 53, 63].

**Figure 1:**
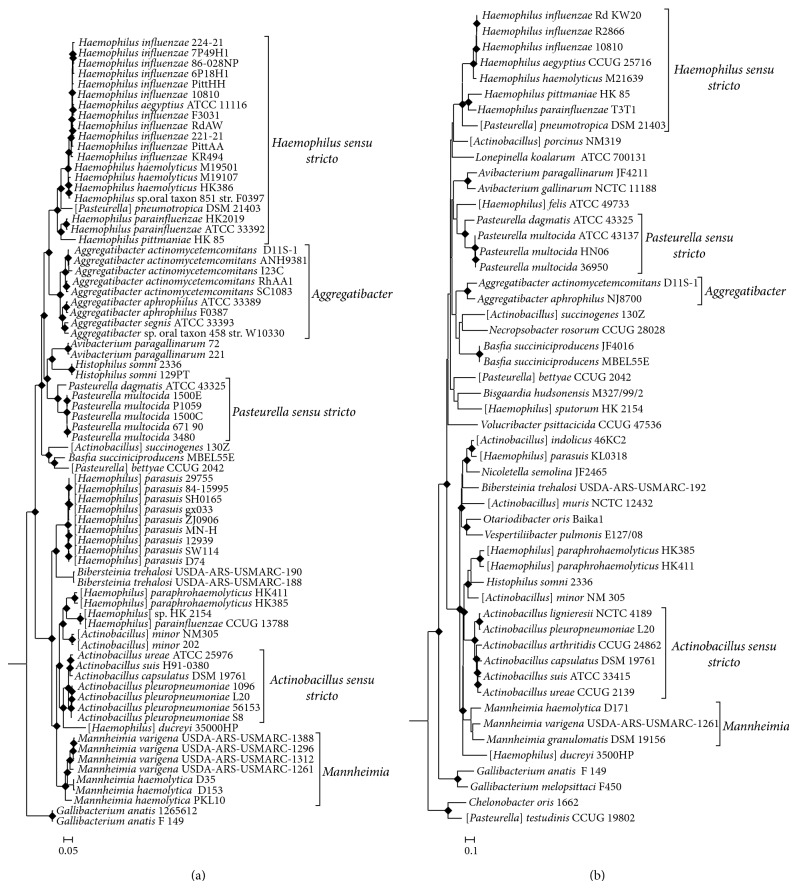
(a) A maximum-likelihood core genome phylogenetic tree of sequenced members of the family* Pasteurellaceae*. (b) A maximum-likelihood phylogenetic tree based on concatenated nucleotide sequence alignments of the 16S rDNA,* infB*,* recN*, and* rpoB* genes. Both trees are rooted using members of the* Vibrionaceae* (not shown). Nodes with >80% bootstrap support are indicated by diamond shaped symbols at the node. Clusters of species representing* Actinobacillus sensu stricto, Haemophilus sensu stricto, Pasteurella sensu stricto*,* Aggregatibacter,* and* Mannheimia* are indicated by brackets. Members of the genera* Actinobacillus, Haemophilus, *and* Pasteurella* which do not fall into their respective “*sensu stricto*” clades are indicated by the presence of square brackets around their generic name (ex. [*Pasteurella*]* pneumotropica*).

Phylogenetic trees based on concatenated sequences for a large number of unlinked and conserved loci are more reliable and robust than phylogenetic trees based on any single gene or protein [64, 65]. Due to a rapid increase in the availability of genomic sequence data, we are now able to complete genome scale phylogenetic analyses of the family* Pasteurellaceae* which cover a vast majority of the diversity within the family. In this work we have produced a phylogenetic tree for 74 genome sequenced members of the family* Pasteurellaceae* based on 128 080 aligned amino acid positions (Figure 1(a)). The branching patterns of the core genome phylogenetic tree produced in this work largely agree with a previous genome based phylogenetic tree produced for a limited number of* Pasteurellaceae *species [19] and a concatenated protein based phylogenetic tree of the family* Pasteurellaceae *produced by our lab in a previous study [20]. Additionally, we have also produced a multilocus sequence based phylogenetic tree using the 16S rDNA,* infB*,* recN*, and* rpoB* genes which are commonly used in the phylogenetic analysis of the family* Pasteurellaceae *(Figure 1(b)) [15–17, 29, 30]. This tree also showed broadly similar branching patterns to past multilocus sequence based phylogenetic trees [17, 18] and to our core genome based phylogenetic tree. Both our core genome based and multilocus sequence based phylogenetic trees provide evidence for a division of the* Pasteurellaceae* into at least two higher taxonomic groups (families) which are broadly similar to the two clades of* Pasteurellales* identified in our previous work [20]. A similar division of the family* Pasteurellaceae* into two or more large groups is seen in many other robust multilocus or concatenated protein based phylogenetic trees [17, 19, 53, 63]; however, this division is not readily apparent in phylogenies based on the 16S rRNA gene [9, 66].

A majority of the known genera within the family* Pasteurellaceae* form well-defined and coherent clusters in phylogenetic trees (Figure 1) [9, 17, 19, 20, 66]. The genera* Actinobacillus, Haemophilus, *and* Pasteurella*, which were described before the advent of genetic characterization, exhibit polyphyletic branching in all gene and protein based phylogenetic trees, including the core genome based and multilocus sequence based phylogenetic trees created in this work (Figure 1). However, there are large clusters of* Actinobacillus, Haemophilus, *and* Pasteurella* species identifiable in the phylogenetic trees which represent the core or “*sensu stricto*” members of each genera. The clusters of species that represent* Actinobacillus sensu stricto, Haemophilus sensu stricto, *and* Pasteurella sensu stricto* are indicated in Figure 1. Members of each genera which fall outside of the* sensu stricto* clusters, indicated in our phylogenetic trees by the presence of square brackets around their genus name (e.g., [*Pasteurella*]* pneumotropica*), are only distantly related to the* sensu stricto* members of their genus and will require reclassification in order to make their taxonomy and phylogeny concordant.

### 3.2. The Usefulness of Conserved Signature Indels as Phylogenetic and Taxonomic Markers

Whole genome sequences are a rich resource for the discovery of molecular signatures which are unique to a group of organisms [67–69]. One useful class of shared molecular signatures are conserved signature indels (CSIs), which are insertions/deletions uniquely present in protein sequences from a group of evolutionarily related organisms [59, 70, 71]. The unique, shared presence of multiple CSIs by a group of related species is most parsimoniously explained by the occurrence of the genetic changes that resulted in these CSIs in a common ancestor of the group, followed by vertical transmission of these CSIs to various descendant species [59, 71–73]. Hence, these CSIs represent molecular synapomorphies (markers of common evolutionary decent) which can be used to identify and demarcate specific bacterial groups in molecular terms and for understanding their interrelationships independently of phylogenetic trees [59, 70–72]. CSIs have recently been used to propose important taxonomic changes for a number of bacterial groups (namely, Aquificae, Spirochaetes, Thermotogae,* Xanthomonadales*, and* Borrelia*) at different taxonomic ranks [69, 74–77]. In the present work, we have completed comprehensive comparative analysis of* Pasteurellaceae* genomes (Table 1) in order to identify CSIs that are primarily restricted to the different genera within the family* Pasteurellaceae*. We have identified 3, 7, and 6 unique molecular signatures which are shared by* Actinobacillus sensu stricto, Haemophilus sensu stricto, *and* Pasteurella sensu stricto*, respectively. Information regarding these CSIs and their evolutionary significances is discussed below.

### 3.3. Molecular Signatures Specific for* Actinobacillus sensu stricto*

**Figure 2:**
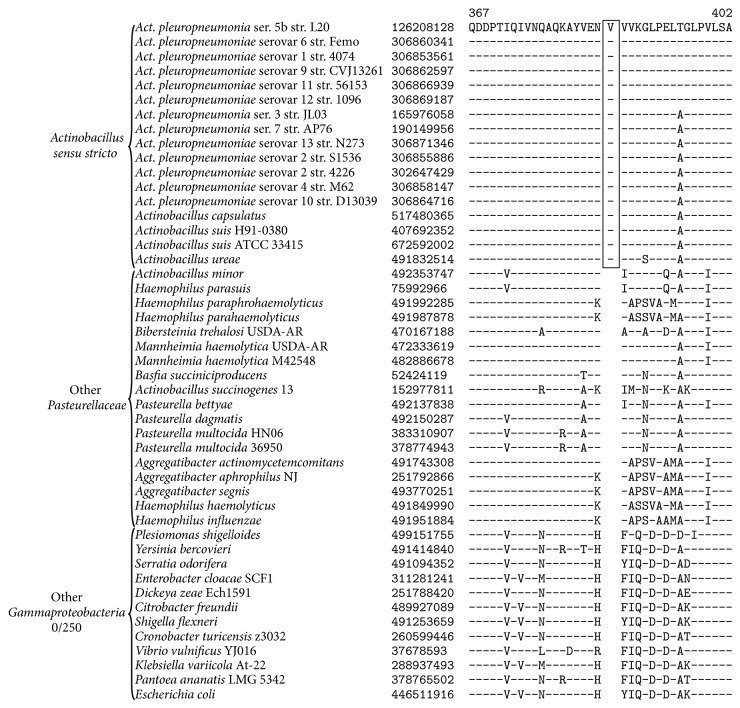
A partial sequence alignment of a 3′-nucleotidase showing a 1-amino-acid insertion identified in all members of* Actinobacillus sensu stricto*. This insertion was not found in the homologues from any member of the genus* Actinobacillus *that was not part of the “*sensu stricto*” clade or any other member of the* Gammaproteobacteria*. Sequence information for a representative subset of the family* Pasteurellaceae* and the class* Gammaproteobacteria* is shown, but unless otherwise indicated, similar CSIs were detected in all members of the indicated group and not detected in any other bacterial species in the top 250 BLAST hits. The dashes (-) in the alignments indicate identity with the residue in the top sequence. GenBank identification (GI) numbers for each sequence are indicated in the second column. Sequence information for other CSIs specific to* Actinobacillus sensu stricto* are presented in Supplemental Figures  1-2 and their characteristics are summarized in Table 2(A).

The genus* Actinobacillus* was originally defined as a group of growth factor independent host-associated rods which shared phenotypic or biochemical similarity with* Actinobacillus lignieresii*, the type species of the genus [24, 78]. However, the original classification scheme for the genus* Actinobacillus *led to the inclusion of a highly heterogeneous and polyphyletic grouping of species within the genus [12–14]. An assemblage of* Actinobacillus* species closely related to* Actinobacillus lignieresii* has been recognized as* Actinobacillus sensu stricto* (i.e., the core members of the genus* Actinobacillus*) in both our phylogenetic analysis (Figure 1) and past phylogenetic analyses [12–14, 17]. Differentiation of* Actinobacillus sensu stricto* from other* Actinobacillus* species and the modern criteria for placing novel species within the genus* Actinobacillus sensu stricto* is heavily reliant on genetic and genomic criteria, namely, DNA-DNA hybridization values, 16S rRNA sequence similarity, and other single gene sequence comparisons [13, 18]. There are currently no known discrete characteristics which are unique to* Actinobacillus* that define the genus. In this work, we have completed a comprehensive comparative analysis of* Pasteurellaceae* genomes in order to identify unique, defining molecular signatures for different genera within the family* Pasteurellaceae*. We have identified 3 CSIs which are unique, defining molecular signatures for the sequenced members of* Actinobacillus sensu stricto *(namely,* Actinobacillus capsulatus, A. pleuropneumoniae, A. suis, *and* A. ureae*). An example of a CSI specific for* Actinobacillus sensu stricto* is shown in Figure 2. The CSI consists of a 1-amino-acid insertion in a conserved region of a 3′-nucleotidase which is present in all sequenced members of* Actinobacillus sensu stricto* and absent in all other sequenced* Gammaproteobacteria*. Sequence information for 2 other CSIs which are also unique characteristics of the* Actinobacillus sensu stricto* clade is presented in Supplemental Figures 1-2 available online at http://dx.doi.org/10.1155/2015/198560 and their characteristics are briefly summarized in Table 2(A).

### 3.4. Molecular Signatures Specific for* Haemophilus sensu stricto*

The classification of novel species into the genus* Haemophilus* was initially based on phenotypic and biochemical properties, most importantly, the dependence of growth on the presence of factor V and factor X in blood [13, 78, 79]. As with* Actinobacillus*, the classification of* Haemophilus* on the basis of phenotypic and biochemical properties has led to the genus containing an extremely heterogeneous group of species [12–14, 32]. Species from the genus* Haemophilus* have undergone a number of transfers and reclassifications [21–24, 28]. However, the genus remains highly polyphyletic (Figure 1) [17, 19, 28]. The core members of the genus* Haemophilus* (namely,* Haemophilus sensu stricto*) consist of* Haemophilus influenzae, H. aegyptius*, and* H. haemolyticus* based on 16S rRNA sequence analysis [12–14, 32]. However, phylogenetic analysis based on DNA-DNA hybridization and multilocus sequence analysis suggests that* H. parainfluenzae *and* H. pittmaniae* are also members of* Haemophilus sensu stricto* [30, 80]. Phylogenetic analysis of* rpoB*,* infB*, and concatenated gene sets also suggest that [*Pasteurella*]* pneumotropica *and related isolates are closely related to* Haemophilus sensu stricto* [15, 16].

**Figure 3:**
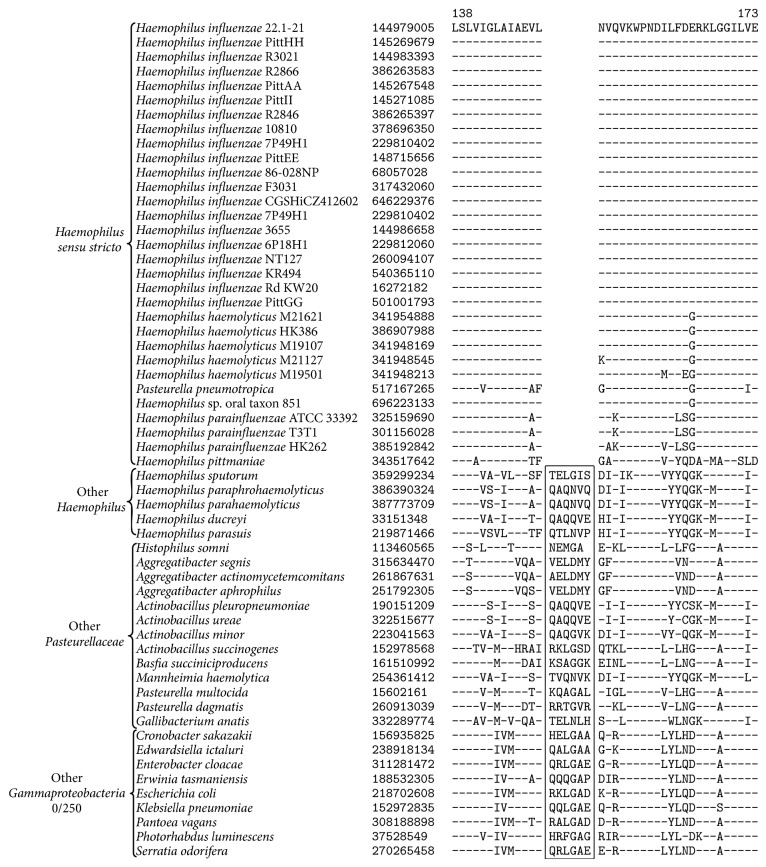
A partial sequence alignment of 1,4-dihydroxy-2-naphthoate octaprenyltransferase showing a 2-amino-acid insertion identified in all members of* Haemophilus sensu stricto*. This insertion was not found in the homologues from any member of the genus* Haemophilus *that was not part of the “*sensu stricto*” clade or any other member of the* Gammaproteobacteria*. Sequence information for other CSIs specific to* Haemophilus sensu stricto* is presented in Supplemental Figures  3–8 and their characteristics are summarized in Table 2(B).

Our comparative analysis of* Pasteurellaceae* genomes has led to the identification of 7 CSIs that are unique characteristics of* Haemophilus sensu stricto* which consists of* Haemophilus influenzae, H. aegyptius, H. haemolyticus, H. parainfluenzae, H. pittmaniae*, and [*Pasteurella*]* pneumotropica *(Figure 1). One example of a CSI specific for the members of* Haemophilus sensu stricto*, shown in Figure 3, consists of a 4-amino-acid deletion in a biotin-protein ligase which is uniquely found in homologs from* Haemophilus sensu stricto* and absent in all other sequenced* Gammaproteobacteria*. Sequence information for 6 additional CSIs which are also unique characteristics of* Haemophilus sensu stricto* is presented in Supplemental Figures  3–8 and their characteristics are briefly summarized in Table 2(B). These CSIs and our phylogenetic trees (Figure 1) suggest that* Haemophilus influenzae, H. aegyptius, H. haemolyticus, H. parainfluenzae, H. pittmaniae*, and [*Pasteurella*]* pneumotropica *share a close evolutionary relationship and should all be considered members of* Haemophilus sensu stricto*. Additionally, these results also suggest that [*Pasteurella*]* pneumotropica* is incorrectly classified as a member of the genus* Pasteurella* and should be reclassified as “*Haemophilus pneumotropica.*”

### 3.5. Molecular Signatures Specific for* Pasteurella sensu stricto*

**Figure 4:**
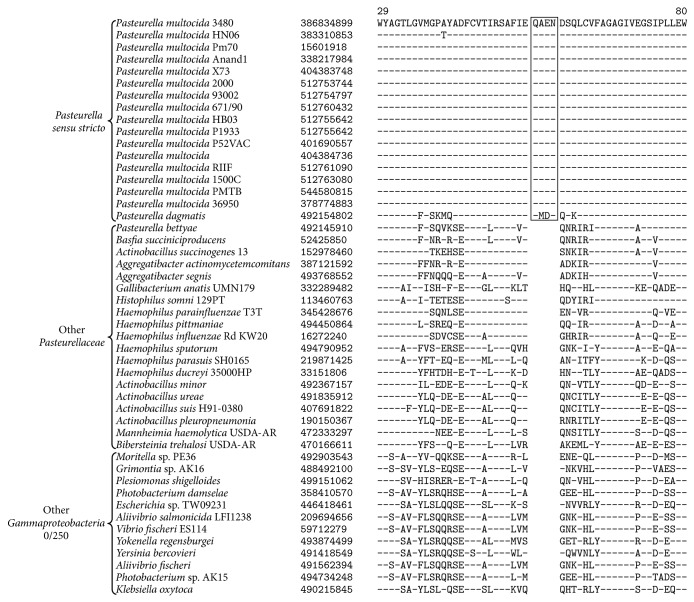
A partial sequence alignment of Menaquinone-specific isochorismate synthase showing a 4-amino-acid insertion identified in all members of* Pasteurella sensu stricto*. This insertion was not found in the homologues from any member of the genus* Pasteurella* that was not part of the “*sensu stricto*” clade or any other member of the* Gammaproteobacteria*. Sequence information for other CSIs specific to* Pasteurella sensu stricto* is presented in Supplemental Figures  9–13 and their characteristics are summarized in Table 2(C).

The genus* Pasteurella* is highly heterogeneous and polyphyletic (Figure 1) [13]. Similar to the members of* Actinobacillus*, bacterial isolates were originally classified as members of the genus* Pasteurella* based on growth factor independent growth and phenotypic or biochemical similarity to* Pasteurella multocida*, the type species of the genus [78, 81]. The monophyletic clusters of* Pasteurella *species that branch with* Pasteurella multocida* are considered the core members of the genus (namely,* Pasteurella sensu stricto*) [9, 13, 16, 17]. Our comparative analysis of* Pasteurellaceae* genomes has led to the identification of 6 CSIs which are unique characteristics for the sequenced members of* Pasteurella sensu stricto *(namely,* Pasteurella multocida *and* P. dagmatis*). An example of a CSI uniquely found in the sequenced members of* Pasteurella sensu stricto*, consisting of a 4-amino-acid insertion in a conserved region of Menaquinone-specific isochorismate synthase, is shown in Figure 4. This CSI is only found in the sequenced members of* Pasteurella sensu stricto* and is absent from all other sequenced* Gammaproteobacteria*. Partial sequence alignments for 5 additional CSIs which are also unique characteristics of* Pasteurella sensu stricto* are presented in Supplemental Figures 9–13 and their characteristics are briefly summarized in Table 2(C).

### 3.6. Molecular Signatures Specific for the Genera* Aggregatibacter* or* Mannheimia*

The genus* Aggregatibacter* was proposed as a novel taxonomic classification for a monophyletic cluster of* Actinobacillus *and* Haemophilus* species which branched distinctly from the “*sensu stricto*” members of their respective clades [21]. Similarly, the genus* Mannheimia* was proposed as a novel classification for the* Pasteurella Haemolytica* complex which did not branch with* Pasteurella sensu stricto* in phylogenetic trees [7]. Currently other than branching in phylogenetic trees or relatedness in DNA-DNA hybridization studies, the members of the genera* Aggregatibacter* or* Mannheimia* do not share any single unique or defining biochemical or molecular characteristic that can differentiate them from all other bacteria [5, 82].

**Figure 5:**
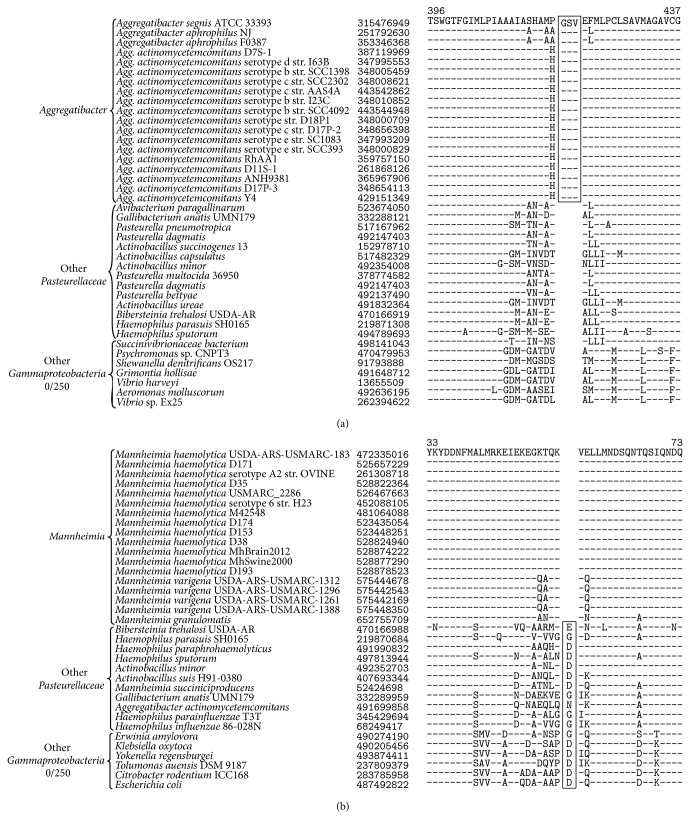
A partial sequence alignment of (a) a* nhaC* family sodium:proton antiporter containing a 3-amino-acid insertion specific for all sequenced species of the genus* Aggregatibacter* (b) a methyl-galactoside ABC transporter substrate-binding protein containing a 1-amino-acid deletion specific for all sequenced species of the genus* Mannheimia*. In each case, the identified CSIs were only found in the sequenced members of the genera* Aggregatibacter* or* Mannheimia* and were absent from all other sequenced* Gammaproteobacteria*. Sequence information for other CSIs specific to* Aggregatibacter* or* Mannheimia *is presented in Supplemental Figures  14–19 and their characteristics are summarized in Tables 2(D) and 2(E).

In this study we have identified 4 CSIs that are unique molecular characteristics shared by all sequenced species of the genus* Aggregatibacter* and another 4 CSIs which are uniquely found in all sequenced members of the genus* Mannheimia*. Examples of CSIs specific to the sequenced members of* Aggregatibacter* and* Mannheimia* are shown in Figure 5. A partial sequence alignment of a* nhaC* family sodium:proton antiporter containing a 3-amino-acid insertion specific for all sequenced species of the genus* Aggregatibacter* is shown in Figure 5(a) and a partial sequence alignment of a methyl-galactoside ABC transporter substrate-binding protein containing a 1-amino-acid deletion specific for all sequenced species of the genus* Mannheimia* is shown in Figure 5(b). In each case, the identified CSIs were only found in the sequenced members of the genera* Aggregatibacter* or* Mannheimia* and were absent from all other sequenced* Gammaproteobacteria*. Partial sequence alignments additional CSIs specific for the genera* Aggregatibacter* or* Mannheimia* are provided in Supplemental Figures 14–19 and their characteristics are summarized in Tables 2(D)-2(E). These CSIs are the first discrete molecular characteristics which are unique for the genera* Aggregatibacter* and* Mannheimia* and support their observed monophyly in phylogenetic trees. Additionally, these CSIs could be useful targets for the development of PCR based diagnostic assays for the genera* Aggregatibacter* and* Mannheimia* which amplify the CSI containing DNA segment using the conserved flanking regions of the CSIs [83, 84].

## 4. Conclusion

**Figure 6:**
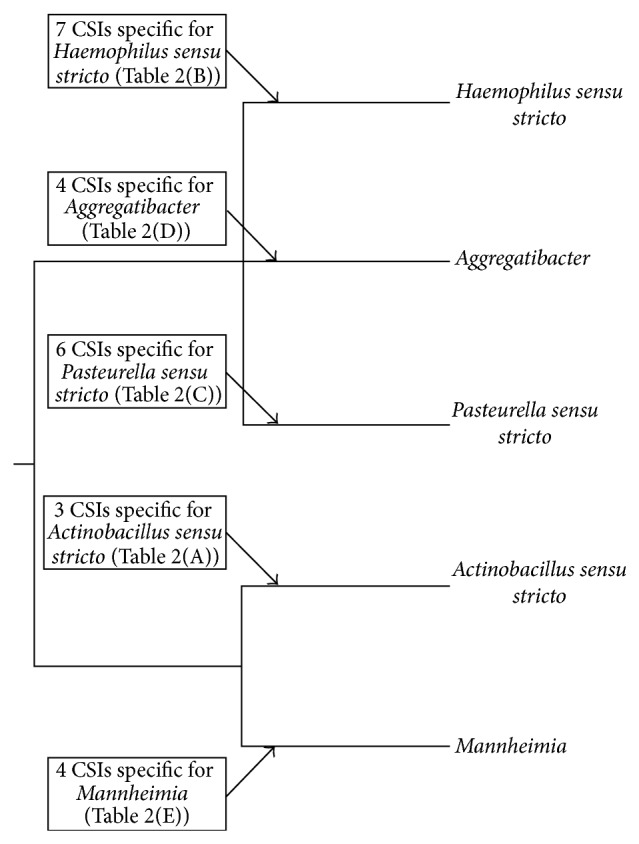
A summary diagram depicting the distribution of identified CSIs for genera within the family* Pasteurellaceae*.

The genera* Actinobacillus, Haemophilus, *and* Pasteurella*, within the family* Pasteurellaceae*, are known to exhibit extensive polyphyletic branching. We have utilized molecular signatures and phylogenetic analyses to clarify the taxonomic boundary of these genera. We have been able to identify large clusters of* Actinobacillus, Haemophilus, *and* Pasteurella* species which represent the “*sensu stricto*” members of these genera. We have identified 3, 7, and 6 unique molecular signatures which are specifically shared by the members of the genera* Actinobacillus sensu stricto, Haemophilus sensu stricto, *and* Pasteurella sensu stricto*, respectively. The group specificity of the molecular signatures we have identified in this work is summarized in Figure 6 and their characteristics are briefly summarized in Table 2. Our comparative genomic analyses have not come across any CSIs that were unique characteristics of all sequenced members of the genera* Actinobacillus, Haemophilus, *or* Pasteurella* as currently defined, suggesting that the members of these genera that do not fall into the “*sensu stricto*” clusters should not be considered members of their respective genus.

Examinations of phenotypic and biochemical characteristics do not provide a reliable means of assigning a novel isolate to the genera* Actinobacillus, Haemophilus, *and* Pasteurella* [18]. However, based upon the CSIs described in this work, it is now possible to demarcate the genera* Actinobacillus sensu stricto, Haemophilus sensu stricto, *and* Pasteurella sensu stricto* on the basis of the presence or absence of unique molecular signatures. It is important to note that the current analysis of CSIs is limited to the currently available genomic sequence data and may show slight variance as additional bacterial genomes are sequenced. However, earlier work on CSIs for other groups of bacteria provides evidence that the identified CSIs have strong predictive value and will likely be found in other members of these groups as more species are sequenced and novel species are isolated [74, 77, 85, 86]. The conserved nature of the sequence regions that contain these CSIs, in conjunction with their strong predictive value, makes CSIs promising targets for the development of highly specific diagnostic assays for* Actinobacillus sensu stricto, Haemophilus sensu stricto, Pasteurella sensu stricto*,* Aggregatibacter,* and* Mannheimia* [83, 84]. Additionally, further analysis of these genus specific CSIs should lead to the discovery of their functional role in their respective organisms and may provide important insights into novel distinguishing features of these groups of organisms.

## Supplementary Material

Partial sequence alignments of the CSI containing regions that were unique characteristics of the genera *Actinobacillus sensu stricto, Haemophilus sensu stricto, Pasteurella sensu stricto, Aggregatibacter*, or *Mannheimia*.

## Figures and Tables

**Table 1:** Genome characteristics of the sequenced *Pasteurellaceae* included in our analyses.

Organism name	BioProject	Size (Mb)	Proteins	G-C (%)	References
*Actinobacillus pleuropneumoniae* L20	CP000569	2.27	2013	41.3	[40]
*Actinobacillus pleuropneumoniae *serovar 3 str. JL03	CP000687	2.24	2036	41.2	[41]
*Actinobacillus pleuropneumoniae *serovar7 str. AP76	CP001091	2.35	2142	41.2	STHH^b^
*Actinobacillus ureae *ATCC 25976^a^	AEVG0	2.30	2475	—	BCM^g^
*Actinobacillus minor *202^a^	ACFT0	2.13	2050	39.3	McGill University
*Actinobacillus minor *NM305^a^	ACQL0	2.43	2411	39.3	McGill University
*Actinobacillus succinogenes *130Z	CP000746	2.32	2079	44.9	Joint Genome Institute

*Aggregatibacter aphrophilus *NJ8700	CP001607	2.31	2219	42.2	[19, 42]
*Aggregatibacter actinomycetemcomitans *D11S-1	CP001733	2.20	2280	44.3	[43]
*Aggregatibacter actinomycetemcomitans *D7S-1	CP003496	2.31	2250	44.3	[44]
*Aggregatibacter segnis *ATCC 33393^a^	AEPS0	1.99	1956	—	BCM^g^

*Gallibacterium anatis *UMN179	CP002667	2.69	2500	39.9	[45]

*Haemophilus aegyptius *ATCC 11116^a^	AFBC0	1.92	2020	—	BCM^g^
*Haemophilus ducreyi *35000HP	AE017143	1.70	1717	38.2	Ohio State University
*Haemophilus haemolyticus *M21621^a^	AFQQ0	2.09	1894	—	[46]
*Haemophilus influenzae *10810	FQ312006	1.98	1903	38.1	WTSI^h^
*Haemophilus influenzae *F3031	FQ670178	1.99	1770	38.2	[47]
*Haemophilus influenzae *F3047	FQ670204	2.01	1786	38.2	[47]
*Haemophilus influenzae *22.1-21^a^	AAZD0	1.89	2224	38.0	[48]
*Haemophilus influenzae *3655	AAZF0	1.88	1929	38.0	[48]
*Haemophilus influenzae *6P18H1^a^	ABWW0	1.91	1893	38.2	CGS, ASRI^e^
*Haemophilus influenzae *7P49H1^a^	ABWV0	1.83	1752	37.9	CGS, ASRI^e^
*Haemophilus influenzae *NT127^a^	ACSL0	1.87	1809	38.0	BIGSP^c^
*Haemophilus influenzae *PittAA^a^	AAZG0	1.88	1981	38.1	[48]
*Haemophilus influenzae *PittII^a^	AAZI0	1.95	2028	38.0	[48]
*Haemophilus influenzae *PittHH^a^	AAZH0	1.84	1977	38.0	[48]
*Haemophilus influenzae *R3021^a^	AAZJ0	1.88	2307	37.9	[48]
*Haemophilus influenzae *RdAW^a^	ACSM0	1.80	1718	38.0	BIGSP^c^
*Haemophilus influenzae *86-028NP	CP000057	1.91	1792	38.2	[49]
*Haemophilus influenzae *PittEE	CP000671	1.81	1613	38.0	[48]
*Haemophilus influenzae *PittGG	CP000672	1.89	1661	38.0	[48]
*Haemophilus influenzae *Rd KW20	L42023	1.83	1657	38.2	[34]
*Haemophilus influenzae *R2846	CP002276	1.82	1636	38.0	UW-SBRI^d^
*Haemophilus influenzae *R2866	CP002277	1.93	1795	38.1	UW-SBRI^d^
*Haemophilus parainfluenzae *ATCC 33392^ a^	AEWU0	2.11	2010	—	BCM^g^
*Haemophilus parainfluenzae *T3T1	FQ312002	2.09	1975	39.6	WTSI^h^
*Haemophilus parasuis *29755^a^	ABKM0	2.22	2244	39.8	Iowa State University
*Haemophilus parasuis *SH0165	CP001321	2.27	2021	40.0	[50]
*Haemophilus pittmaniae *HK 85^a^	AFUV0	2.18	2390	—	J. Craig Venter Institute
*Haemophilus sputorum *CCUG13788^a^	AFNK0	2.14	2073	—	Aarhus University Hospital
*Haemophilus parahaemolyticus *HK385^a^	AJSW0	1.81	1764	—	J. Craig Venter Institute
*Haemophilus paraphrohaemolyticus *HK411^a^	AJMU0	2.02	2025	—	J. Craig Venter Institute
*Haemophilus sp. oral taxon 851 *str.F0397^a^	AGRK0	1.84	1809	—	GCG-WU^f^

*Histophilus somni *2336	CP000947	2.26	1980	37.4	Joint Genome Institute
*Histophilus somni *129PT	CP000436	2.01	1798	37.2	[51]

*Mannheimia succiniciproducens *MBEL55E	AE016827	2.31	2370	42.5	[52]
*Mannheimia haemolytica* PHL213^a^	AASA0	2.57	2695	41.1	[53]

*Pasteurella multocida *subsp*. multocida *str*. Pm70 *	AE004439	2.26	2012	40.4	[54]
*Pasteurella dagmatis *ATCC 43325^a^	ACZR0	2.25	2053	37.4	BCM^g^

^a^The genomes of these species/strains are currently under scaffolds/contigs status.

^b^Stiftung Tieraerztliche Hochschule Hannover (STHH).

^c^The Broad Institute Genome Sequencing Platform (BIGSP).

^d^University of Washington; Seattle Biomedical Research Institute (UW-SBRI).

^e^Center for Genomic Sciences, Allegheny-Singer Research Institute (CGS, ASRI).

^f^Genome Sequencing Center (GSC) at Washington University (WashU) School of Medicine.

^g^Baylor College of Medicine (BCM).

^h^Wellcome Trust Sanger Institute (WTSI).

**Table 2:** Conserved signature indels specific for genera within the family *Pasteurellaceae*.

Protein name	Gene name	GenBank identifier	Figure number	Indel size	Indel position^a^
(A) CSIs specific for *Actinobacillus sensu stricto *					
3′-nucleotidase	*surE *	126208128	Figure 2	1 aa ins	367–402
GTP pyrophosphokinase	*relA *	126207889	Sup. Figure 1	1 aa ins	368–412
Anaerobic glycerol-3-phosphate dehydrogenase subunit	*glpA *	491834528	Sup. Figure 2	1 aa ins	359–400

(B) CSIs specific for *Haemophilus sensu stricto *					
Biotin-protein ligase	*birA *	144979005	Figure 3	6 aa del	138–178
Aspartate ammonia-lyase	*aspA *	145630289	Sup. Figure 3	1 aa ins	34–75
NAD(P) transhydrogenase subunit alpha	*pntA *	145631394	Sup. Figure 4	1 aa del	352–378
Fumarate reductase subunit C	*frdC *	301169552	Sup. Figure 5	3 aa ins	31–89
Hypothetical tRNA/rRNA methyltransferase	—	145636352	Sup. Figure 6	1 aa del	17–58
Gamma-glutamyl kinase	*proB *	145629980	Sup. Figure 7	1 aa ins	197–253
ACP phosphodiesterase	*acpD *	68250119	Sup. Figure 8	2 aa del	119–159

(C) CSIs specific for *Pasteurella sensu stricto *					
Menaquinone-specific isochorismate synthase	*menF *	386834899	Figure 4	4 aa ins	29–86
tRNA s(4)U8 sulfurtransferase	*thiI *	15602400	Sup. Figure 9	2 aa del	412–446
FKBP-type peptidyl-prolyl cis-trans isomerase	*slyD *	378775595	Sup. Figure 10	2 aa del	151–188
Aspartate-semialdehyde dehydrogenase	*asd *	383311492	Sup. Figure 11	1 aa del	173–245
Lactate permease family transporter	*lldP *	492154065	Sup. Figure 12	2 aa ins	390–427
Cell division protein *ftsA *	*ftsA *	492155843	Sup. Figure 13	1 aa ins	357–387

(D) CSIs specific for *Aggregatibacter *					
*nhaC* family sodium:proton antiporter	*nhaC *	493769836	Figure 5(a)	3 aa ins	396–437
Outer membrane protein	*omp *	261866907	Sup. Figure 14	4 aa del	25–64
Multidrug transporter *murJ *	*murJ *	365966332	Sup. Figure 15	1 aa del	190–220
NADH dehydrogenase	*nuoE *	387120244	Sup. Figure 16	1 aa ins	372–412

(E) CSIs specific for *Mannheimia *					
Methyl-galactoside ABC transporter substrate-binding protein	—	472335016	Figure 5(b)	1 aa del	33–73
UDP-N-acetylmuramoylalanyl-D-glutamate–2,6-diaminopimelate ligase	*murE *	472333011	Sup. Figure 17	2 aa del	418–473
Glutathione-regulated potassium-efflux protein	*kefC *	472333189	Sup. Figure 18	1 aa ins	504–531
Glycerol-3-phosphate acyltransferase	*plsB *	472334521	Sup. Figure 19	2 aa del	214–252
