# Supplementary material for: Phylogenomic and Molecular Demarcation of the Core Members of the Polyphyletic Pasteurellaceae Genera Actinobacillus, Haemophilus, and Pasteurella
Source: Int J Genomics. 2015 Mar 3;2015:198560. doi: 10.1155/2015/198560 (PMC4363679; doi:10.1155/2015/198560)
Supplement: Supplementary file 1 — Partial sequence alignments of the CSI containing regions that were unique characteristics of the genera Actinobacillus sensu stricto, Haemophilus sensu stricto, Pasteurella sensu stricto, Aggregatibacter, or Mannheimia. [file 198560.f1.pdf]

			368		412
Actinobacillus sensu stricto	Act pleuropneumonia ser. 5b str. L20	126207889	WLRKLLAWQNDIADSGEIMA	A	EMRSQVFDDRVYVFTPKGEVIDLP
	Act pleuropneumoniae ser 6 str. Femo	306860648	--------------------	-	------------------------
	Act pleuropneumonia ser. 5b str. 5b str. L20	126096681	--------------------	-	------------------------
	Act pleuropneumoniae ser 2 str. 4226	302648616	--------------------	-	------------------------
	Act pleuropneumoniae ser 2 str. S1536	306856105	--------------------	-	------------------------
	Act pleuropneumoniae ser 4 str. M62	306858326	--------------------	-	------------------------
	Act pleuropneumoniae ser 10 str. D13039	306865081	--------------------	-	------------------------
	Act pleuropneumoniae ser 12 str. 1096	306869447	--------------------	-	------------------------
	Act pleuropneumoniae ser 13 str. N273	306871633	--------------------	-	------------------------
	Act pleuropneumoniae ser 1 str. 4074	306853881	--------------------	-	------------------------
	Act pleuropneumoniae ser 9 str. CVJ13261	306862884	--------------------	-	------------------------
	Act pleuropneumoniae ser 11 str. 56153	306867306	--------------------	-	------------------------
	Act pleuropneumonia ser. 7 str. AP76	190149698	--------------------	-	--S---------------------
Other *Pasteurellales*	Pasteurella pneumotropica	517167434	---------D--T----V--		-L------------------V---
	Actinobacillus capsulatus	517481243	----------------DVV-		----------------R-------
	Avibacterium paragallinarum	545595425	---------D--T--S-VI-		-L----------------------
	Actinobacillus suis H91-0380	407692614	----------------DVV-		----------------R-------
	Actinobacillus ureae	491834566	----------------DVV-		----------------R-------
	Actinobacillus minor	492371309	----------------DVV-		--------------------V---
	Actinobacillus succinogenes 13	152977877	---------A------G-VG		---T-I--------------V---
	Mannheimia succiniciproducens	52424296	---------H--SN---VVP		-L-T----------------V---
	Pasteurella bettyae	492134420	---------H--S-A--VVP		-L-T--------------Q-----
	Pasteurella multocida 36950	378775075	---------D--T-------		------------------------
	Pasteurella multocida subsp. m	15603730	---------D--T-------		------------------------
	Pasteurella dagmatis	492150615	---------D--T----V--		------------------------
	Mannheimia haemolytica USDA-AR	472334407	------E----L----DMV-		-----I----------R-------
	Bibersteinia trehalosi USDA-AR	470167385	-----------------MVD		DL----------------------
	Haemophilus ducreyi 35000HP	33152293	-----------L-V-SDM--		D----I----------R-------
	Haemophilus sputorum	494789823	---------------SDMVD		--------------------V---
	Haemophilus parahaemolyticus	491987266	----------------DVLD		----------------R-------
	Haemophilus paraphrohaemolytic	491989861	----------------DVVD		----------------R-------
	Haemophilus parasuis SH0165	219870950	------D--K------DVV-		--------------------V---
	Haemophilus somnus 129PT	113460244	---------D--T----V--		--------------------V---
	Haemophilus parainfluenzae T3T	345428882	---------D--T----V--		-L------------------V---
	Haemophilus sp. oral taxon 851	497184571	---------D--T----V--		-L------------------V---
	Haemophilus haemolyticus	491876336	---------D--T----V--		-L------------------V---
	Haemophilus influenzae R2846	386265268	---------D--T----V--		-L------------------V---
	Haemophilus influenzae 10810	378696520	---------D--T----V--		-L------------------V---
	Haemophilus influenzae Rd KW20	16272286	---------D--T----VL-		-L------------------V---
	Haemophilus influenzae PittEE	148825382	---------D--T----VL-		-L------------------V---
	Haemophilus influenzae 86-028N	68248939	---------D--T----VL-		-L------------------V---
	Haemophilus influenzae F3047	319775796	---------D--T----VL-		-L------------------V---
	Haemophilus influenzae F3031	319896667	---------D--T----VL-		-L------------------V---
	Haemophilus influenzae PittGG	148827481	---------D--T---KV--		-L------------------V---
	Haemophilus pittmaniae	494450183	---------D--T----VIE		-L------------------V---
	Haemophilus aegyptius	494054052	---------D--T----VL-		-L------------------V---
	Aggregatibacter actinomycetemc	261867309	---------D--T----V--		-L------------------V---
	Aggregatibacter segnis	493770955	---------D-------V--		-L------------------V---
	Aggregatibacter aphrophilus NJ	251793350	---------D--T----V--		-L------------------V---
	Gallibacterium anatis UMN179	332289404	---------D-L-----VL-		------------------------
Other *Gammaproteobacteria* 0/250	Psychromonas ingrahamii 37	119944303	----I----E-----EDLVD		-V----------------------
	Xenorhabdus nematophila	498909906	-----I---EEM--T--MLD		-V----------------D-----
	Serratia symbiotica	493761013	-----IT--EEM-----MLD		-V----------------D-V---
	Alteromonas sp. SN2	333894384	---RI----EEV-E--DMVE		-L----------------D-----
	Arsenophonus nasoniae	284009170	-----I---EEM-----MLD		-V----------------D-----
	Sodalis glossinidius str. 'mor	85058488	---------EEM-----MLD		-V----------------D-V---
	Cronobacter sakazakii ATCC BAA	156932730	-----I---EEM-----MLD		-L----------------D-V---
	Morganella morganii subsp. mor	455738312	-----I---EEM-----MLD		-V----------------D-V---
	Salmonella enterica	487688971	-----I---EEM-----MLD		-V----------------D-V---
	Shigella flexneri	491272795	-----I---EEM-----MLD		-V----------------D-V---
	Citrobacter freundii	489124323	-----I---EEM-----MLD		-V----------------D-V---
	Escherichia coli	446148970	-----I---EEM-----MLD		-V----------------D-V---

Supplementary Figure 1

Partial sequence alignment for GTP pyrophosphokinase. The protein contains 1 aa insertion that is specific for *Actinobacillus sensu stricto.*

Group	Organism	Accession	359		400
Actinobacillus sensu stricto	Actinobacillus ureae ATCC 25976	322119155	TITGGKLMTYRLMAEWATDLVCKKLNKSDR	L	CSTAERPLPGS
	Actinobacillus suis H91-0380	407692608	----------------T-------------	-	-----------
	Actinobacillus suis ATCC 33415	672592320	----------------T-------------	-	-----------
	Actinobacillus capsulatus	517481249	------------------------------	-	-----------
	Act. pleuropneumonia ser. 3 str. JL03	165975838	------------------------------	-	-----------
	Act. pleuropneumonia ser. 6 str. Femo	302651308	------------------------------	-	-----------
	Act. pleuropneumonia ser. 2 str. 4226	302648590	------------------------------	-	-----------
	Act. pleuropneumonia ser. 12 str. 1096	306869420	------------------------------	-	-----------
	Act. pleuropneumonia ser. 4 str. M62	306858454	------------------------------	-	-----------
	Act. pleuropneumonia ser. 2 str. S1536	306856077	------------------------------	-	-----------
	Act. pleuropneumonia ser. 10 str.D13039	306865053	------------------------------	-	-----------
	Act. pleuropneumonia ser. 13 str. N273	306871605	------------------------------	-	-----------
	Act. pleuropneumonia ser. 1 str. 4074	306853852	------------------------------	-	-----------
	Act. pleuropneumonia ser. 9 str. CVJ13261	306862854	------------------------------	-	-----------
	Act. pleuropneumonia ser. 11 str. 56153	306867274	------------------------------	-	-----------
	Act. pleuropneumonia ser. 5b str. L20	126207863	------------------------------	-	-----------
	Act. pleuropneumonia ser. 7 str. AP76	190149672	------------------------------	-	-----------
Other *Pasteurellales*	Actinobacillus minor	492378962	------------------------------		-T---------
	Actinobacillus succinogenes 13	152977891	--------------------------VNKK		-E--D-T----
	Mannheimia succiniciproducens	52308100	----------------------Q---N-KK		-E-SD-T----
	Pasteurella bettyae	492141813	--------------------------N-KT		-E--D-V----
	Pasteurella dagmatis	492153891	---------------------------NAE		-V--KQA----
	Pasteurella multocida subsp. m	15603307	---------------------------HAE		-V--TQ-----
	Pasteurella multocida 36950	378775293	---------------------------HAE		-V--TQ-----
	Gallibacterium anatis UMN179	332289452	--------------------I-----HQAE		-K--KQ-----
	Bibersteinia trehalosi USDA-AR	470167686	-------------------------G-TE-		-T-H-------
	Haemophilus ducreyi 35000HP	33152271	---------------------------VS-		-I---CA----
	Haemophilus parasuis SH0165	219871479	---------------------------T--		-V---KA----
	Haemophilus pittmaniae	494450103	---------------------------TA-		-T--D------
	Haemophilus parainfluenzae T3T	345429343	---------------------------TA-		-T---------
	Haemophilus influenzae F3031	319897870	---------------------------TV-		-T---------
	Haemophilus influenzae Rd KW20	16272627	---------------------------TA-		-V---Q-----
	Haemophilus influenzae PittGG	148827859	---------------------------TA-		-V---Q-----
	Haemophilus influenzae 86-028N	68249264	---------------------------TA-		-V---Q-----
	Haemophilus influenzae R2846	386266588	---------------------------TA-		-V--KQ-----
	Haemophilus influenzae F3047	319775464	---------------------------TA-		-V---QA----
	Haemophilus influenzae 10810	378696883	---------------------------TA-		-V---QA----
	Haemophilus influenzae PittEE	148826678	---------------------------TA-		-V---QA----
	Haemophilus haemolyticus	491864144	----------------------E----TA-		-V---Q-----
Other *Gammaproteo-bacteria* 0/250	Plesiomonas shigelloides	499149535	-----------------S----R---H-AT		-T---K-----
	Citrobacter rodentium ICC168	283786321	-------------------A--R--GN-RP		-V--DT-----
	Serratia marcescens FGI94	440228953	------------------N---E--GV-A-		-T---QA----
	Morganella morganii subsp. mor	455740577	-------------------A--A--GITAH		-----E-----
	Enterobacter asburiae LF7a	345300129	--------------Q----A--R--GNTEP		-V--DK-----
	Klebsiella oxytoca	491003182	-------------------A--R--GN-VP		----DT-----
	Raoultella ornithinolytica B6	481848065	-------------------A--R--GN-VP		----DT-----
	Yersinia pestis	488309597	-------------------K--E--GI-AA		-T--QV-----
	Escherichia coli	486207164	-------------------A--R--GN-RP		-T--DLA----
	Shigella flexneri	491270753	-------------------A--R--GNTRP		-T--DLA----
	Vibrio cholerae O395	147671420	---------------------A---GNTQS		-Q-HL------

Supplementary Figure 2

Partial sequence alignment for anaerobic glycerol-3-phosphate dehydrogenase subunit A. The protein contains 1 aa insertion that is uniquely shared by *Actinobacillus sensu stricto.*

Group	Species	ID	34		75
Haemophilus Sensu Stricto	Haemophilus influenzae PittEE	148825193	INDLLPEDAIDPEYYDKLLQTL	Q	NDAGIPVLDEAPESDDMML
	Haemophilus influenzae 22.1-21	144979387	----------------------	-	-------------------
	Haemophilus influenzae 86-028NP	68249130	----------------------	-	-------------------
	Haemophilus influenzae 3655	144985884	----------------------	-	-------------------
	Haemophilus influenzae KR494	543951383	----------------------	-	-------------------
	Haemophilus influenzae R2866	491910936	----------------------	-	-------------------
	Haemophilus influenzae F3047	503292748	----------------------	-	-------------------
	Haemophilus influenzae 10810	505740084	----------------------	-	-------------------
	Haemophilus sp. oral taxon 851	497182876	--------V-------------	-	---------------E---
	Haemophilus haemolyticus M21639	491862772	--------V-------------	-	---------------E---
	Haemophilus haemolyticus M21127	491851008	--------V-------------	-	---------------E---
	Haemophilus haemolyticus M19107	491841414	--------V-------------	-	---------------E---
	Haemophilus haemolyticus M19501	491847886	--------V-------------	-	---------------E---
	Haemophilus parainfluenzae	503830655	--------L-------------	-	H--------------E---
	Pasteurella pneumotropica	517167701	--------V-------------	-	H---------P------I-
	Haemophilus pittmaniae	343519915	--------V-------------	-	---------------E---
Other *Haemophilus*	H. sputorum	359299231	VH-H---ELV-A-QIEDII-MI		T-M--Q-FEV--DT-EL--
	H. paraphrohaemolyticus	386390298	VH-H---ELV-A-QIEDII-MI		T-M--Q--ET--DT-EL--
	H. parahaemolyticus	387772261	VH-H---ELV-A-QIEDII-MI		T-M--K--ET--DT-EL--
	H. ducreyi	33151868	VH-H---ELV-A-QIEDII-MI		--M--K--ET--DT-EL--
	H. parasuis	167855425	VH-H---ELV-ADQIEDII-MI		--M--K--ET--DA--L--
	Histophilus somni	170719016	V--H----LV-ADQIEDVI-MI		--M--Q--ESV-DA--L--
Other *Pasteurellales*	Act. pleuropneumoniae	307264154	VH-H---ELV-A-QIEDII-MI		--M--K--ET--DT-EL--
	Act. ureae	322513554	VH-H---ELV-A-QIEDII-MI		--M--K--ET--DT-EL--
	Act. succinogenes	152978401	V--H----LV-TDQIEDII-MI		--M--Q--ET--DA--LL-
	Act. minor	257464575	VH-H---ELV-A-QIEDII-MI		T-M--Q--ET--DT-EL--
	Agg. actinomycetemcomitans	261868195	V--S---ELV-ADQIEDII-MI		--M--Q--ET--DA--L--
	Agg. segnis	315634867	V--S---ELV-ADQIEDII-MI		--M--Q--ET--DA--L--
	Agg. aphrophilus	251793982	V--S---ELV-ADQIEDII-MI		--M--Q--ET--DA--L--
	Man. succiniciproducens	52425815	V--H---ELV-TDQIEDII-MI		--M--Q--ES--DA--L--
	Man. haemolytica	261492895	VH-H---ELV-A-QIEDII-MI		--M--Q--EA--DT--LL-
	Past. multocida	383311804	V--H---ELV-A-QIEDII-MI		--M--Q--ES--DA--L--
	Past. dagmatis	260912696	V--H---ELV-A-QIEDII-MI		--M--Q--ET--DA--L--
	Gallibacterium anatis	332288976	V--S----LV-ADQIEDII-MI		--M--Q--ES--DA--L--
Other *Gammaproteobacteria* 0/250	Acinetobacter lwoffii	262376664	V--H--DSITES-QIEDII-M-		--V----H-R------T-F
	Aliivibrio salmonicida	209696105	V--H---EIV-S-QVEDII-MI		--M--K-VET--DA---S-
	Arsenophonus nasoniae	284008589	V--H----IV-SDQIEDII-MI		--M--Q-ME---DA--LI-
	Baumannia cicadellinicola	94676896	V-------IV-SDQIEDII-MI		--M--Q-ME---DA--IL-
	Buchnera aphidicola	384227506	V--H----I--S-QI-DII-MI		--M----VE---DA--LI-
	Citrobacter freundii	365103141	V--H----IV-SDQIEDII-MI		--M--Q-ME---DA--LL-
	Congregibacter litoralis	88704608	V--H----IS--DQVEDII-MI		--M--Q-FEQ--DA--LI-
	Cronobacter turicensis	260599325	V--H----IV-SDQIEDII-MI		--M--Q-ME---DA--LL-
	Dickeya dadantii	307129360	V--H----I--SDQIEDII-MI		--M--Q-ME---DA--L--
	Edwardsiella ictaluri	238918478	V--H----IV-SDQIEDII-MI		--M--Q-ME---DA--L--
	Enterobacter aerogenes	336247240	V--H----IV-SDQIEDII-MI		--M--Q-ME---DA--LL-
	Erwinia amylovora	292486903	V--H----IV-SDQIEDII-MI		--M--Q-VE---DA--L--
	Escherichia coli	377982750	V--H----IV-SDQIEDII-MI		--M--Q-ME---DA--L--
	Grimontia hollisae	262275021	V--H----IV-SDQIEDII-MI		--M--Q-VET--DA-EL-M
	Klebsiella oxytoca	376381681	V--H----IV-SDQIEDII-MI		--M--Q-ME---DA--LL-
	Oceanimonas sp. GK1	374336959	V--H--Q-IV-SDQIEDII-MI		--M--Q-VEN--DA--L-M
	Pantoea agglomerans	390435428	V--H----IV-SDQIEDII-MI		--M--Q-VE---DA--L--
	Photorhabdus luminescens	37527834	V--H----IV-SDQIEDII-MI		--M--Q-ME---DA--L--
	Proteus penneri	226327186	V--H----IV-SDQIEDII-MI		--M--Q-ME---DA--L--
	Providencia stuartii	183597880	V--H----IV-SDQIEDII-MI		--M--Q-ME---DA--LI-
	Rahnella sp. Y9602	322831339	V--H----IV-SDQIEDII-MI		--M--Q--E---DA--L--
	Salmonella enterica	213161043	V--H----IV-SDQIEDII-MI		--M--Q-ME---DA--LL-
	Serratia odorifera	270263179	V--H----IV-SDQIEDII-MI		--M--Q-ME---DA--LL-
	Shewanella baltica	386340136	V--H--A-MV-SDQIEDII-MI		--M--R-FE---DA----M
	Shigella boydii	187733221	V--H----IV-SDQIEDII-MI		--M--Q-ME---DA--L--
	Sodalis glossinidius	85058229	V--H----IV-SDQIEDII-MI		--M--Q-ME---DA--LL-
	Vibrio fischeri	371493750	V--H---EIV-S-QVEDII-MI		--M--K-VET--DA---S-
	Xenorhabdus bovienii	290474860	V--H----IV-SDQIEDII-MI		--M--Q-ME---DA--L--
	Yersinia aldovae	238760057	V--H----IV-SDQIEDII-MI		--M--Q--E---DA--L--
	Yokenella regensburgei	365847769	V--H----IV-SDQIEDII-MI		--M--Q-ME---DA--LL-

Supplementary Figure 3

Partial sequence alignment for Aspartate ammonia-lyase. The protein contains 1 aa (glutamine) insertion that is specific for a clade consisting of *H. influenzae, H. aegyptius, H. haemolyticus, H. sp. oral taxon 851, H. parainfluenzae,* and *H. pittmaniae*. The remaining *Haemophilus* species and other *Pasteurellales* and *Gammaproteobacteria* species (250 blasts) do not contain the CSI.

			352		378
Haemophilus Sensu Stricto	Haemophilus influenzae 3655	144986549	DVVLRGVTVVRDGEEI		PPAQIQVSAQP
	Haemophilus influenzae R3021	491888782	----------------		-----------
	Haemophilus influenzae 10810	505740607	----------------		-----------
	Haemophilus influenzae KR494	543997178	----------------		-----------
	Haemophilus influenzae PittGG	500630890	----------------		-----------
	Haemophilus influenzae PittEE	500625845	----------------		-----------
	Haemophilus influenzae 22.1-21	491882181	----------------		-----------
	Haemophilus sp. FF7	692226876	----------------		-----------
	Haemophilus influenzae Rd KW20	16273272	----------------		-----------
	Pasteurella pneumotropica	517167151	----------------		-----------
	Haemophilus pittmaniae	494451436	----------------		-----------
	Haemophilus haemolyticus M19107	491841549	----------------		-----------
	Haemophilus haemolyticus M21127	491853823	----------------		-----------
	Haemophilus parainfluenzae HK262	491967783	----------------		-----------
	Haemophilus parainfluenzae ATCC 33392	491966042	----------------		-----------
	Haemophilus parainfluenzae T3T1	503831535	----------------		-----------
	Haemophilus aegyptius	494053950	----------------		-----------
	H. sp. oral taxon 851	373467219	----------------		-----------
Other *Haemophilus*	H. parasuis	167854584	---------I---KVT	W	-APP-------
	H. parahaemolyticus	387773029	---------I-E--VT	W	-APP-------
	H. sputorum	359299851	---------I----LT	W	-APP-------
	H. paraphrohaemolyticus	386389589	-----------E--VT	W	-APP-------
	H. ducreyi	33152693	---V-----I----VT	W	-APP-------
	Histophilus somni	113460378	---------I----VT	W	-APP------T
Other *Pasteurellales*	Act. ureae	322514553	--------------LT	W	-APP-------
	Act. pleuropneumoniae	190150168	--------------LT	W	-APP-------
	Act. succinogenes	152978693	---------I----VT	W	-APP-------
	Act. minor	257465417	---------I----VT	W	-APP-------
	Agg. segnis	315634400	---------I----VT	W	-APP-------
	Agg. actinomycetemcomitans	293390251	---------I---KIT	W	-APP-------
	Agg. aphrophilus	251792709	---------I-E--IT	W	-APP-------
	Gallibacterium anatis	332288590	---Q-----I----VT	W	-APP-K-----
	Man. haemolytica	254361623	--------------LT	W	-APP------L
	Man. succiniciproducens	52425279	---I-----I-N--VT	W	-APP-K-----
	Past. multocida	15602618	---------I----VT	W	-APP-------
	Past. dagmatis	260914246	---------I-E--VT	W	-APP-K-----
Other *Gammaproteobacteria* 0/250	Aliivibrio salmonicida	209809408	----------KE--VT	W	-APP-------
	Enterobacter cloacae	311279548	---V-----I-E--VT	W	-APP-------
	Escherichia coli	187776147	---I-----I-A--IT	W	-APP-------
	Grimontia hollisae	262273996	----------K---IT	W	-APP-------
	Pantoea ananatis	291617548	---I----------VT	W	-APP-----A-
	Pectobacterium carotovorum	227111338	-T-I-----IKE--IT	W	-APP-------
	Photobacterium profundum	90412639	-E----L--IKE--IT	W	-APP-K---A-
	Photorhabdus luminescens	37526083	---I------KE--MT	W	-APP-------
	Proteus penneri	226329945	---I-----I-A--VT	W	-APP-------
	Providencia stuartii	183599058	---I-----IKQ--IT	W	-APP-------
	Salmonella enterica	168235558	---I-----I---DIT	W	-APP-------
	Vibrio fischeri	197337488	----------KE--VT	W	-APP-------
	Vibrionales bacterium	148981352	----------KE--VT	W	-APP-------
	Yersinia rohdei	238749352	-T-I------KT--VT	W	-APP-------

Supplementary Figure 4

Partial sequence alignment for NAD(P) transhydrogenase subunit alpha. This protein region show 1 aa (Tryptophan) deletion that is specific for a clade consisting of *H. influenzae, H. aegyptius, H. haemolyticus, H. sp. oral taxon 851, H. parainfluenzae,* and *H. pittmaniae*. The remaining *Haemophilus* species and other *Pasteurellales* or *Gammaproteobacteria* species (250 blasts) do not contain the CSI.

```
                                                      31                                                          89
Haemophilus Sensu Stricto
Haemophilus influenzae 86-028NP  68249428     LREATSVFAVWFCIVLLYGVLCLASNP MPG LGILSFIEFLRNPIVVFLNIITLIATLYH
Haemophilus influenzae PittGG    148828019    --------------------------- --- -----------------------------
Haemophilus influenzae F3031     319897673    --------------------------- --- -----------------------------
Haemophilus influenzae R3021     144983972    --------------------------- --- -----------------------------
Haemophilus influenzae F3031     317433179    --------------------------- --- -----------------------------
Haemophilus influenzae 10810     378697034    --------------------------- --- -----------------------------
Haemophilus influenzae PittHH    145270132    --------------------------- --- -----------------------------
Haemophilus influenzae HK1212    270317146    --------------------------- --- -----------------------------
Haemophilus influenzae M19501    341949445    --------------------------- --- -----------------------------
Haemophilus influenzae M21127    341950834    --------------------------- --- -----------------------------
Haemophilus influenzae M21639    341956009    --------------------------- --- ---------------------------
Haemophilus haemolyticus         342905181    ------I-------------------- I-- -----------------------------
Haemophilus aegyptius            329122840    ------I-------------------- IL- -D---------------------------
Haemophilus parainfluenzae       325579196    V-----I-------------------- V-- ---VD--G---------------------
Haemophilus pittmaniae           343517839    ------I-------------------- --- M--FD--G--K------------V-----
Haemophilus sp. oral taxon 851   373466532    ------I-------------------- I-- -----------------------------
Pasteurella pneumotropica        2516029750   ------I-------------------- I-- H--F------------L------V-----
Other Haemophilus
Haemophilus sputorum             359298516    V-----IPT---SL--F---IT-GRGT      FATD-VS-VQ----IVF-L-S-A-I---
Haemophilus paraphrohaemolyticus 386389927    V----AIPT----L------IA-GNGT      FDTN-VD--K----II--LVS-A-M-L-
Haemophilus parahaemolyticus     387772210    V----AIPT----L------IS-GNGT      FDT--VD--K----II--LVS-A-M-L-
Haemophilus parasuis             167855691    ---S---PT----L--F--LV--GNGT      FATT-VG--Q--V--I------G-V-LN
Haemophilus ducreyi              33151306     I----CIATI---L------IS-GGRH       -EN--S-SQ--L--I----S-AGL---
Histophilus somni                113461422    M----AIPSL--SL------I--GQKD     GLQSD--P--Q----II----A-A-AILN
Other Pasteurellales
Act. minor                       240948013    V----AIPT----L-----LIS-GNGT      FATD-LT--K--LI-IF-LVS-V-M---
Act. ureae                       322513266    V----CLAT----L------IS-GGEG       LEN--S--Q----FI--V-SIA-L---
Act. pleuropneumoniae            46143534     V----CLAT----L---H--IS-GGGS       LDG--S--Q----FI--V-SIA-L---
Act. succinogenes                152979472    V----A-PT----L------FS--D-T      FATD-VG-MQ----MI----S-A-M-L-
Agg. aphrophilus                 251793019    -----ALPT---SL------I--GKAD     GFVMN--P--Q---I-I---VS-A-L-L-
Agg. segnis                      315633841    -----A-PT---SL------I--GKAD     GFVM---P--Q--L--I----S-A-L-L-
Agg. actinomycetemcomitans       261867504    V----ALPT---SL------I--GKAD     GF-A---P--Q-----I----S-A-L-L-
Man. succiniciproducens          152979472    V----A-PT----L------FS--D-T      FATD-VG-MQ----MI----S-A-M-L-
Man. haemolytica                 254362605    V----CLPT-------F--AVA-SKGT      FTTD-VD--Q--L--I----S-A-M---
Past. dagmatis                   260914415    A----AIPTL---L------IS-GNIE      SFGN--A--------I------G-M-LN
Past. multocida                  15602064     A----AIPTL---L------IS-G-LD      SFGN--S--K----II------G-M-LN
Gallibacterium anatis            332288836    ---S---PTI---LE-F--LT--GNGT      FDSN-VN--Q--V--I------G-V-LN
Other Gammaproteobacteria 0/250
Citrobacter koseri               157147874    ---G-A-P----S-E-IF-LFA-KHGA     ESWMG-VG--Q--V--I--L-A-A-A-L-
Cronobacter turicensis           260599495    ---G---P----S---I--LFA-KHG-     ESWAG-VG--Q--V--L------L-A-L-
Dickeya zeae                     251788028    ---G-A-P----S----H--FS-KAG-     E-WGH-VG--Q--L-LL---VA-L-AVL-
Edwardsiella tarda               304557750    ---SSALEN---S---I---FS-KDGA     A-WE--VA--Q--L-LV----S-LLAVL-
Enterobacter aerogenes           336248333    ---G-A-PT---S---I--LFA-KHGA     ESWEGYVA--Q-----I--L---A-A-L-
Erwinia billingiae               300715026    -----ALP-L--SLEMIF-LFA-KNGE     QSWQA-VAV-QH--MLLI--VA-A-A-L-
Escherichia coli                 15804746     ---G-A-P----S-E-IF-LFA-KNG-     EAWAG-VD--Q--VI-II-L---A-A-L-
Grimontia hollisae               262273031    -----VLPLIF-TLC-TF-LGS-VLGE     AAWN-WLS-MA--VAIAI--VA-AGS-F-
Klebsiella pneumoniae            206578658    V--G-A-PT---S---I--LFA-KHGA     ESWAGY-G--Q--V--I--L---A-A-L-
Pectobacterium atrosepticum      50122891     ---G-A-P----S-I-MC-IFA-KNG-     E-WAN-VS--Q--L-LLI--VA-L-AAL-
Photobacterium profundum         54310447     V----ILPLIF-T-C-TF-LG--VKG-     EAWAGWLS-MS-----V---LA-LGS-F-
Providencia alcalifaciens        212710997    M--S---LQ---S-LV----FA-KNG-     ESWAG-VG--S---ILII--V--A---L-
Salmonella enterica              194448129    ---G-A-P----S-E-IF-LFA-KHGA     ESWMG-VG--Q--V--I--L---A-A-L-
Serratia odorifera               293393230    ---G---P----S---I---FA-KGG-     DSWVG--G--Q--L-LLI--VA-L-A-L-
Shigella dysenteriae             82779425     ---G-A-P----S-E-IF-LFA-KNG-     EAWAG-VD--Q--VI-II-L---A-A-L-
Vibrio cholerae                  15642653     V----VLPLIL-TLF-TV-LGS-VKG-     EAWQTWLD-MA--L-IAI-LVA-AGS-F-
Vibrionales bacterium            148976863    -----VLPLIL-TVF-TF-LGA-VKG-     EAWAGWLS-MA---I-GI--VA-LGS-M-
Xenorhabdus nematophila          300721664    ---G---P----SLLV---LFA-KNG-     ESWAG-VA--Q----LLI-----L-A-L-
Yersinia pestis                  22124529     ---G--IP----SVL-I---FA-K-G-     A-WEG-VS--Q--L-L----L--F-A-L-
```

Supplementary Figure 5

Partial sequence alignment for Fumarate reductase subunit C. The protein contains 3 aa insertion that is specific for a clade consisting of *Haemophilus influenzae, Haemophilus aegyptius, Haemophilus haemolyticus, Haemophilus sp. oral taxon 851, Haemophilus parainfluenzae,* and *Haemophilus pittmaniae*. The remaining *Haemophilus* species and other *Pasteurellales* or *Gammaproteobacteria* species (250 blasts) do not contain the CSI.

Group	Species	Accession	17	CSI	58
Haemophilus Sensu Stricto	Haemophilus influenzae 22.1-21	145636352	NIGSAARAMKTMGLTQLCLVSPKS		VDEQSYALSAGAENIVKN
	Haemophilus influenzae PittGG	148827519	------------------------		------------------
	Haemophilus influenzae F3047	319775753	------------------------		------------------
	Haemophilus influenzae F3031	319896709	------------------------		------------------
	Haemophilus influenzae KW20	20178339	------------------------		------------------
	Haemophilus influenzae NT127	260094339	------------------------		------------------
	Haemophilus influenzae R2866	386263423	------------------------		------------------
	Haemophilus influenzae PittII	309750201	------------------------		------------------
	Haemophilus influenzae 10810	378696563	------------------------		------------------
	Haemophilus influenzae PittAA	145268169	------------------------		------------------
	Haemophilus influenzae R2846	386265225	------------------------		------------------
	Haemophilus influenzae 86-028NP	68057182	------------------------		------------------
	Haemophilus influenzae 3655	144986822	------------------------		------------------
	Haemophilus influenzae 7P49H1	229810560	------------------------		------------------
	Haemophilus influenzae 6P18H1	229812781	------------------------		------------------
	Haemophilus influenzae PittHH	145270517	------------------------		------------------
	Haemophilus influenzae Rd KW20	16272328	------------------------		------------------
	Haemophilus haemolyticus	142904025	------------------------		-------------D----
	Haemophilus aegyptius	329123594	------------------------		------------------
	Haemophilus parainfluenzae	301155740	--------------SN-Y------		-----I--A---DDL-R-
	Haemophilus pittmaniae	343519305	--------------NE-Y-----C		I----I--A---DD--I-
	Pasteurella pneumotropica DSM 21403	516029676	--------------S--------V		-----I--A---AD----
	Haemophilus sp. oral taxon 851	373467487	--------------A---------		-------------D----
Other Haemophilus	Haemophilus sputorum	359299850	----V---------H-----A-QK	A	I---AV--A---KDVLD-
	Haemophilus parasuis	167854979	--------------SR-R--A--Q	A	I---AI--A---KDVLD-
	Haemophilus ducreyi	33152694	--------------SN-R--R--N	P	L---AT--A---K--LD-
	Haemophilus paraphrohaemolyticus	386389590	-------------ISH-R--A--Q	P	L--DAF------KDLLE-
	Haemophilus parahaemolyticus	387773034	----V---------SH-R--E--R	P	L--DAF------KDLLE-
Other Pasteurellales	Histophilus somni	113460425	--------------N-----A--Q	G	I---AV--A----DV--S
	Act. pleuropneumoniae	307254675	--------------SN-R----LN	P	I----Q--A---KDVLD-
	Act. minor	240949393	----T---------A--------Q	P	I---AVS-A---KDVLD-
	Act. succinogenes	152978538	---------------N-Y--A---	R	I-A-AV-------DVI--
	Act. ureae	322514554	--------------SN-R----LN	P	I---AQ--A---KDVLD-
	Agg. aphrophilus	251792921	---------------D-V--A--Q	G	--D-AI--A----DL--S
	Agg. actinomycetemcomitans	293390584	---------------D-V--A--Q	S	----AI--A----DV--S
	Agg. segnis	315633898	----T----------D-V--A--Q	G	--D-AI--A----DV--S
	Man. succiniciproducens	52425783	--------------AN-Y-----Q	G	I---AV-------DVLRK
	Man. haemolytica	261493539	---------------H-R--A--Q	P	L---AE--A---KDVLD-
	Past. dagmatis	260912947	--------------SH-Y--A-RQ	A	I---AI-------DV--Q
	Past. multocida	15602181	--------------N--Y--A--Q	P	L--HAI-------DVL-Q
	Gallibacterium anatis	332289843	--------------S--Y-----V	L	PN-E-I------DDV--Q
Other Gammaproteobacteria 0/250	Aeromonas hydrophila	117618072	-M------------S-MV--D-QA	Q	P-DNA---A---SDLLA-
	Alteromonadales bacterium	119470463	--------------SK-Y--D-AC	E	I-SHAS------SDVLG-
	Ferrimonas balearica	308050472	--------------S--F-AE--V	A	P-G--I--A---SDLL--
	Grimontia hollisae	262274801	----------V---KHMV--A-EC	K	--G-AI--A---SD-AN-
	Photobacterium damselae	269103289	----------V----N-V--D-AC	E	I---AM--A---SD--E-
	Proteus penneri	226328027	-M--T----------N-YF-N--E	K	P-SH-I------SDVIG-
	Pseudoalteromonas haloplanktis	332532349	--------------SK-Y--D-AC	E	I-S-AS--A---TDVLG-
	Pseudoxanthomonas suwonensis	319787464	---A----------SR-V----EK	P	L-DTA-RR-----DVLDS
	Psychromonas ingrahamii	119945066	-V-------------K-V--T-EC	E	I-GK-I------SDVL--
	Shewanella loihica	127513248	----T---------ST-Y-AE--V	A	P-G--V--A---SD-L-H
	Shigella dysenteriae	194432193	----V----------N-W--N-LV	K	P-S-AI--A---SDVIG-
	Sodalis glossinidius	85059749	----T---------SN-Y--N-LH	K	P-A-AI------SD-IG-
	Tolumonas auensis	237808766	--------------S--V--D-VQ	P	P--K-T--A---ND-LA-
	Vibrio angustum	90579380	----------V----N-V--D-AC	E	--GHTI--A---ADV-E-
	Vibrionales bacterium	148979594	----------V---S--V--D-QC	E	----TL--A---GD-AE-
	Xanthomonas fuscans	294624425	---A---------VSR-V--A-ER	A	L--DA-RR-----DVLSQ
	Xenorhabdus bovienii	290476021	-M-------------N-Y--N-LV	Q	P-SH-I------SDVIG-
	Yersinia intermedia	238795106	-M--T----------N-Y--N-LV	K	P-S-AI------SDVIG-

Supplementary Figure 6

Partial sequence alignment for Hypothetical tRNA/rRNA methyltransferase. This protein region show 1 aa deletion that is specific for a clade consisting of *Haemophilus influenzae, Haemophilus aegyptius, Haemophilus haemolyticus, Haemophilus sp. oral taxon 851, Haemophilus parainfluenzae,* and *Haemophilus pittmaniae*. The remaining *Haemophilus* species and other *Pasteurellales* or *Gammaproteobacteria* species (250 blasts) do not contain the CSI.

Group	Species	GI	197		253
Haemophilus Sensu Stricto	H. influenzae R3021	144984261	ATRSGIETIIAPGNRPNVIADLAYEQNIGTKFIA	H	QSDRLESRKQWLFAAPSAGIIT
	H. influenzae 3655	144987185	----------------------------------	-	----------------------
	H. influenzae PittAA	145268512	----------------------------------	-	----------------------
	H. influenzae 22.4-21	145273391	----------------------------------	-	----------------------
	H. influenzae 6P18H1	229813038	----------------------------------	-	----------------------
	H. influenzae M19107	341951493	----------------------------------	-	----------------------
	H. influenzae F3047	317449758	----------------------------------	-	----------------------
	H. influenzae M21127	341949145	----------------------------------	-	----------------------
	H. influenzae 10810	378697101	----------------------------------	-	----------------------
	H. influenzae PittHH	145269827	----------------------------------	-	----------------------
	H. influenzae NT127	260095321	----------------------------------	-	----------------------
	H. influenzae R2866	386264654	----------------------------------	-	----------------------
	H. influenzae PittII	145272608	----------------------------------	-	----------------------
	H. aegyptius	329122915	---------------------------------V	-	----------------------
	H. haemolyticus	342903547	-----------------I----------------	-	----------------------
	H. pittmaniae	343517903	---------------A---V----QKP-------	-	-A--------------CV-TL-
	H. parainfluenzae	345429559	-------------S--D--V----DKP-------	-	H------------------ML-
	H. sp. oral taxon 851	373467991	----------------------------------	-	----------------------
	Pasteurella pneumotropica	2516030440	-------------------V---H--G-----T-	-	HA-----------------A--
**Other *Haemophilus***	H. paraphrohaemolyticus	386389256	-----V-----S-S-E--LV---NGHA--S--L-		------G---------QV-S-V
	H. parahaemolyticus	387772439	-----V-----S-S-E--LV---NG-A--S--VV		------G---------QV-S-V
	H. parasuis	167855493	-I---V--A--S-E-----FEV-QGAE---R-L-		-H-KI-G------G--P--T-Y
	H. sputorum	359300234	-----V-----S-S-E--LV---NGKA--S--LV		------G---------QV-S-Y
**Other *Pasteurellales***	Histophilus somni	170718754	-----V--------LE---VN---GKD-----VV		-T---------I----V---V-
	Act. succinogenes	152978396	--------V-------D--T---HGKS-----TV		APEH---------------VVI
	Act. minor	240948719	-----V-----S-S-E--LV---NGKE--S--LV		------G---------QV-S--
	Act. pleuropneumoniae	307254720	-----V--V--S-S-A--LV---NGES--S--LV		------G---------QV-SLV
	Act. ureae	322514098	-----V-----S-S-E--LV---NDVA--S--LV		---C--G---------QV-AL-
	Agg. actinomycetemcomitans	347991576	--------------Q----V------P------V		-A-----------------VL-
	Agg. aphrophilus	347813523	--------------QA---V------A-----TV		-A-----------------VLV
	Agg. segnis	315633625	--------------Q----V----D-A-----TV		-A-----------------VL-
	Man. haemolytica	254361022	-------------------V----G-A-----SV		-A----------Y---------
	Man. succiniciproducens	52307969	---------------E-------HGEA-----TV		-T-K----------------L-
	Past. dagmatis	260912819	---------------S--------GET-------		------------Y---------
	Past. multocida	386835057	-------------------V----G-A-----SV		-A----------Y---------
	Gallibacterium anatis	332289427	-------------G-K---L---NNLA-----TV		PPHAI-----------IT-H--
**Other *Gammaproteobacteria* 0/250**	Arsenophonus nasoniae	284007761	-G-A---V---A-DK-----NVLEG-QV----HC		LKTPM-N--R-I-G--L--E-V
	Citrobacter koseri	157147175	-C-A--D----A-SK-G--G-VMAGISA--R-H-		-ASP--N--R-I-G--P--E--
	Cronobacter sakazakii	156935258	-G-A----V--A-SK-G--N-VIEGLPV--R-HP		-QTP--N--R-I-G--P--E--
	Dickeya dadantii	307132275	-C----DV---A---TG----VINDVSV--R-H-		LETP-----R-I-G--P--E--
	Edwardsiella ictaluri	238918850	-C-A---VV--A-S--G--GEVIDGISV--R-HP		LQTP--N--R-I-G--P--E-V
	Enterobacter aerogenes	336248914	-C-A--D----A----D--GHAMEGLPV--C-H-		-ESP--N--R-I-G--P--E--
	Erwinia amylovora	292487375	-C-A--D----A-S--G--G-AIACNPV--R-H-		LETP--N--R-I-G--P--ELV
	Escherichia coli	15799947	-C-A--D----A-SK-G--G-VMEGISV--L-H-		-ATP--N--R-I-G--P--E--
	Hafnia alvei	365837494	-C-A--DV---A-SK-G-VG-VIDGVSV--R-H-		LETP--N--R-I-G--P--E--
	Klebsiella oxytoca	376388964	-C-A--D----A-S--G--G-VMEGVSV--R-H-		-ESP--N--R-I-G--P--E--
	Pantoea ananatis	291616445	-C-A--DV---A-S-SG--S-VIAGKPV--L-H-		-QTP--N--R-I-G--P--E--
	Photorhabdus asymbiotica	253990692	-G-A--DV-V-A-SKS---S-VIEGNPV--R-HG		LASPM-N--R-I-G--P--E-I
	Proteus mirabilis	227357828	-G-A--DVV--A-VK-D--SKVIDNEPV--L-HG		LKSP--T--R-I-G--V--V-I
	Providencia alcalifaciens	212709386	-G-A--DV---A--K-D--NAVIENIPV--R-YG		-KNPM-N--R-I-G--A--DVY
	Rahnella aquatilis	383188939	-C-A-VD----A-SL-G--G-VINGVSV--R-H-		METP--N--R-I-G--P--E--
	Salmonella enterica	56414551	-C-A--D----S-SK-G--G-VMEGISV--R-H-		-ASP--N--R-I-G--P--E--
	Serratia marcescens	130967	-C-A--DVV--A-SK-G-V--VIEGKPV--R-H-		LETP--N--R-I-G--P--E--
	Shigella dysenteriae	320176707	-C-A--D----A-SK-G--G-VMEGISV--L-H-		-ATP--N--R-I-G--PG-E--
	Sodalis glossinidius	85058578	-N-A---VV--A-GK-G--G-MMAGASV--R-HG		-KMP-----R---GP-P--D--
	Xenorhabdus bovienii	290476274	-G-A-VDV---A----E-V--VIEGKSV--R-HG		-ACPM-N--R-I-G--P--E-I
	Yersinia aldovae	238760030	-C-A--DVV--A-SQVG----VIDGTPV--R-H-		LETP--T--R-I-G--P--E--
	Yokenella regensburgei	365848145	-C-A--D----A-S--G--G-VMEGVSV--R-H-		-ASP--N--R-I-G--P--E--

Supplementary Figure 7

Partial sequence alignment for Gamma-glutamyl kinase. The protein contains 1 aa (Histidine) insertion that is specific for a clade consisting of *H. influenzae, H. aegyptius, H. haemolyticus, H. sp. oral taxon 851, H. parainfluenzae,* and *H. pittmaniae*. The remaining *Haemophilus* species and other *Pasteurellales* or *Gammaproteobacteria* species (250 blasts) do not contain the CSI. *Haemophilus ducreyi* do not contain the homologue protein.

Group	Organism	ID	119		159
Haemophilus Sensu Stricto	Haemophilus influenzae HK1212	270317394	GSEGLLKGKKAIVLCAFGGLYDEE		NLVTQYMKSILGFIGIT
	Haemophilus influenzae 86-028NP	68250119	------------------------		-----------------
	Haemophilus influenzae R2846	386265814	------------------------		-----------------
	Haemophilus influenzae KR494	543952153	------------------------		-----------------
	Haemophilus influenzae 3655	144986545	------------------------		-----------------
	Haemophilus influenzae 6P18H1	229813169	------------------------		-----------------
	Haemophilus influenzae 7P49H1	229810271	------------------------		-----------------
	Haemophilus influenzae R2866	386264219	------------------------		-----------------
	Haemophilus influenzae 22.1-21	144978396	------------------------		-----------------
	Haemophilus influenzae PittHH	145270596	------------------------		-----------------
	Haemophilus aegyptius	329123507	-----------F------------		-----------------
	Haemophilus haemolyticus	342904212	-P----------------------		---------V-------
	Haemophilus pittmaniae	343519865	-P-----D------A-C--M---D		-N--N-L-A--D-V---
	Haemophilus parainfluenzae	325578420	-P----Q-------AS---M---N		-N--N-L-A----V---
	Haemophilus sp. oral taxon 851	373467223	-P----------------------		-----------------
	Pasteurella pneumotropica DSM 21403	2516029975	-P--------------------D-		--------A----V---
Other *Haemophilus*	Haemophilus parahaemolyticus	387772242	-----IT----V-ILTY--I-KDG	LA	D-PKL--QTV-N-----
	Haemophilus paraphrohaemolyticus	386389687	-P---IT------IL-H--I-KDS	PT	D-AKL--QTV-N-----
	Haemophilus parasuis	167856647	-P---I-----V-VL-H--I-KDG	PA	D-AKM--QTV-N-----
Other *Pasteurellales*	Agg. segnis	315634175	-P---------V--A----FHQNQ	PT	D---S---A----V---
	Agg. aphrophilus	251792472	-P----T----V--A----FHQNQ	PT	D---A-V-T--------
	Agg. actinomycetemcomitans	293390341	-P----T----V--A----FHQNQ	SS	DI--A-L-M----V---
	Past. dagmatis	260914079	-P---I-----L-ILTS--IHQGS	AT	D--AA---IM-------
	Gallibacterium anatis	332288876	-PK---PNVPVFAFVTS--E-EGT	EF	DTMSDWV-QV---V-LS
Other *Gammaproteo-bacteria* 0/250	Cronobacter sakazakii	156933866	-P---VT--R----TSR--IHKDT	PT	D---P-LSLF-------
	Dickeya dadantii	271500288	-P---V---R-V--TSR--IHKDT	ES	DV-AP-LRLM-------
	Enterobacter cloacae	296102444	-P---V---R----TSR--IHKDT	PT	D--AP--SLF-------
	Enterobacteriaceae bacterium	317491990	-P--------VY--TSR--IHKDT	DT	D---P-LRVF-----L-
	Erwinia pyrifoliae	259908386	-P---I----VV--SSR--IHKDT	PS	D-L-P-LTLF---L-MS
	Escherichia coli	324117609	-P---VT------ITSR--IHKDG	PT	D---P-LSTF----S--
	Grimontia hollisae	262275640	--Q---T----LIVTTR--MHVGQ	SS	DHI-G-L-TV---V---
	Pantoea ananatis	291617560	-P---V---R-VI-SSR--IHKDS	PS	D-L-P-V-LF-------
	Photorhabdus luminescens	37526065	-P-------QV-I-TSR--IHKDG	PS	D-LVP-LRLF-S---L-
	Serratia odorifera	270262050	-P---V---R--I-TSR--IHKGT	PT	D--EP-LRLF-------
	Shigella boydii	320178234	-P---VT------ITSR--IHKDG	PT	D---P-LSTF-------
	Vibrio harveyi	153831886	-VQ--IE------VTTR---HKDS	PT	DSM-P-LRTV---V---
	Xenorhabdus bovienii	290475378	-P-----N-R----TSR--IHKDN	PT	D-I-P-LRTF------N
	Yersinia kristensenii	238762559	-P---IT--R--I-TSR--IHKDT	PT	D--VP-LRLF-------

Supplementary Figure 8

Partial sequence alignment for ACP phosphodieterase. The protein contains 2 aa deletion that is specific for a clade consisting of *Haemophilus influenzae, Haemophilus aegyptius, Haemophilus haemolyticus, Haemophilus sp. oral taxon 851, Haemophilus parainfluenzae,* and *Haemophilus pittmaniae*. The remaining *Haemophilus* species and other *Pasteurellales* or *Gammaproteobacteria* species (250 blasts) do not contain the CSI.

Group	Organism	Accession	412		446
Pasteurella sensu stricto	Past. multocida subsp. m str. Pm70	15602400	DIRSPEETDEKPLNMENV		QLMPFYKLSSQFANLDQ
	Past. multocida subsp. m str. 3480	386834344	------------------		-----------------
	Past. multocida subsp. m str. P52VAC	401689778	------------------		-----------------
	Past. multocida subsp. m str. PMTB	544580593	------------------		-----------------
	Past. multocida subsp.m str.VTCCBAA264	544580593	------------------		-----------------
	Past. multocida subsp. m str. 3480	385201045	------------------		-----------------
	Past. multocida subsp. gallicida	385201045	------------------		-----------------
	Past. multocida subsp. m str. 3480	385201045	------------------		-----------------
	Past. multocida subsp. m P1062	557249467	------------------		-----------------
	Past. multocida subsp. m str. Anand1	338215897	------------------		-----------------
	Past. multocida subsp. m str. HN06	383310178	------------------		-----------------
	Past. multocida 1500C	512763580	------------------		-----------------
	Pasteurella multocida RIIF	512762374	------------------		-----------------
	Pasteurella multocida 93002	512757128	------------------		-----------------
	Pasteurella multocida 671/90	512759585	------------------		-----------------
	Pasteurella multocida 36950	378774244	------------------		-----------------
	Pasteurella multocida 1500E	511371788	------------------		-----------------
	Pasteurella multocida 2000	512752228	------------------		-----------------
	Pasteurella multocida P1933	512756828	------------------		-----------------
	Pasteurella dagmatis	492146744	----------N--K-D--		K-----------ST---
Other *Pasteurellales*	Pasteurella pneumotropica	517166719	------------FKSDHH	EV	IQ----------SS---
	Pasteurella bettyae	492137292	-------H--R-FSL-GY	EV	KHL---------ST-P-
	Mannheimia succiniciproducens	52425117	-------H--R-FELAGH	EV	KHL---------GD---
	Actinobacillus succinogenes 13	152979406	----------N--AL--R	QV	IEL----V----GE---
	Avibacterium paragallinarum	516417163	-------S--N--QLA-A	KI	-------------E---
	Aggregatibacter segnis	493769782	-------I--N--HI--Q	AM	I-L----------E---
	Aggregatibacter aphrophilus NJ	251792601	-------I--N--HI--Q	AM	I-L------G---D---
	Aggregatibacter actinomycetemc	365967873	-------I-----HI-KQ	TM	I-L------N---E---
	Gallibacterium anatis UMN179	332288104	-------HE-E--QLD-H	TI	LHI------T--PS---
	Haemophilus somnus 129PT	113461045	-------V-D---EISGQ	NI	I---------H--E---
	Haemophilus somnus 2336	170717619	-------V-D---EISGQ	NI	I---------H--E---
	Actinobacillus capsulatus	517481949	----------T-FSLDG-	EV	K------------S---
	Actinobacillus suis H91-0380	407691879	----------A-FSLDG-	EV	K------------S---
	Actinobacillus pleuropneumonia	491785160	----------A-FSLDG-	EV	K------------S---
	Actinobacillus ureae	491831251	----S-----A-FSLDG-	EV	K------------S---
	Actinobacillus minor	492375229	-------V----FSL-G-	EV	KEL---------GA---
	Bibersteinia trehalosi USDA-AR	470166787	----------N-FELDG-	NV	-I-------NN-GS---
	Mannheimia haemolytica USDA-AR	472333421	-------V----FRLDG-	EV	KEL---------ST---
	Haemophilus parasuis SH0165	219870478	-------V----FSL-G-	EV	KEL------NH-G----
	Haemophilus parahaemolyticus	491983005	----------A--ELD--	EV	KEL---------TA---
	Haemophilus paraphrohaemolytic	491988803	----------A-FKL-G-	EV	KEL---------TE---
	Haemophilus ducreyi 35000HP	33152814	-------M-----VLAQA	QV	IEL------T---H---
	Haemophilus sputorum	494789029	--------E-S-FTL-G-	TV	KVL---------GS---
	Haemophilus influenzae 86-028N	68250032	------------FESGTH	DV	IQ----------GS---
	Haemophilus aegyptius	494053895	----------N-FESGTH	DV	IQ----------GS---
	Haemophilus sp. oral taxon 851	497183028	----------N-FESDTH	EV	IQ----------GS---
Other *Gammaproteo-bacteria* 0/250	Candidatus Regiella insecticol	493756535	---A---QE----DLD	PV	HIL-----R---ST---
	Yersinia rohdei	490854420	---A-D-Q-----KL-GI	EV	-AI----------D---
	Dickeya dadantii 3937	307129908	-----D-Q--R--AL-Q-	EI	KSL-----GT--GD---
	Plesiomonas shigelloides	499151317	-------EE----VL-G-	TV	TC-------T--DT---
	Enterobacteriaceae bacterium s	440288781	----VD-Q-D---KV-G-	EI	ASL------T--G----
	Salmonella enterica	486431237	----VD-Q-D---KV-G-	DV	VSL------TK-GD---
	Brenneria sp. EniD312	496405054	---A-D-QE----TLAQA	EI	KSL------T--GD---
	Serratia marcescens	491078757	----ND-QE----QL-Q-	EV	KAL------T--GD---
	Shewanella sp. W3-18-1	120599623	---A---EEH----I-G-	EV	KRI--F--AT---D--K
	Pantoea sp. GMO1	495162200	----AD-Q-A---AL-G-	EV	KPLA-----T--GD---
	Colwellia psychrerythraea 34H	71280272	-------EED---ELGD-	EV	KHI------T--GD--
	Kosakonia radicincitans	494615276	----ID-Q-D---KL-G-	DV	VSL------TK-GD---

Supplementary Figure 9

Partial sequence alignment for tRNA s(4)U8 sulfurtransferase. The protein contains 2 aa deletion that is specific for *Pasteurella sensu stricto.*

Group	Organism	Accession	151		188
Pasteurella sensu stricto	Pasteurella multocida 36950	378775595	DTVTVNYTGKLPNGEVFDSS		RGKPVSFKLNQVVPGWTE
	Past. multocida subsp. m str. HN06	383311604	--------------------		------------------
	Past. multocida subsp. m str. Anand1	338218483	--------------------		------------------
	Past. multocida subsp. m str. gallicida	338219570	--------------------		------------------
	Past. multocida subsp. m str. Anand1	401693313	--------------------		------------------
	Past. multocida subsp. m str. 3480	385202348	--------------------		------------------
	Past. multocida subsp. m str.P52VAC	401688712	--------------------		------------------
	Past. multocida subsp. m str. PMTB	544580133	--------------------		------------------
	Past. multocida subsp. m str.P1062	557250232	--------------------		------------------
	Past. multocida subsp.m VTCCBAA264	662151453	--------------------		------------------
	Pasteurella multocida 3695	356598143	--------------------		------------------
	Pasteurella multocida 1500E	511371593	--------------------		------------------
	Pasteurella multocida 2000	512753119	--------------------		------------------
	Pasteurella multocida 671/ 90	512758749	--------------------		------------------
	Pasteurella multocida 1500C	512764075	--------------------		------------------
	Past. multocida subsp. m str. HB03	570283202	--------------------		------------------
	Pasteurella multocida 93002	512757188	--------------------		------------------
	Past. multocida subsp. m str. Pm70	15603214	--------------------		------------------
	Pasteurella dagmatis	492150696	-----K-V------D---RT		H---I-------------
	Past. dagmatis ATCC 43325	260632528	-----K-V------D---RT		H---I-------------
Other *Pasteurellales*	Haemophilus ducreyi	33151282	-I-IAH-K-T--D-T-----	YE	-NE-IELQ-K-LI-A-I-
	Haemophilus sputorum	498362100	---K-H-K-T-VD-S-----	YD	--Q-IE---DNLI-A-K-
	Bibersteinia trehalosi	575453520	---K-H-K-T-TD-S-----	YE	--E-IE-Q-S-LI-A-V-
	Gallibacterium anatis	332288995	---K---R---ID-TE----	YK	-NQ-IE-Q-G-LI---V-
	Haemophilus paraphrohaemolyticus	491991260	-V-K-H-K-T--D-T-----	YD	-NA--E-Q-D-LI-A-V-
	Haemophilus parahaemolyticus	386907676	-M-K-H-K-T--D-T-----	YD	-NA--E-Q---LI---I-
	Actinobacillus minor	257450706	-I-K-H-K-T----TT----	YD	--E-IE-Q-D-LI---I-
	Mannheimia haemolytica	544866063	---K-H-K-T-TD-T-----	YD	--E-IE-Q---LI---I-
	Actinobacillus capsulatus	517481536	---K-H-K-T-TD-T-----	YD	--E-IE-Q---LI---I-
	Actinobacillus pleuropneumoniae	491828212	---K-H-K-T-TD-T-----	YD	--E-IE-Q---LI---I-
	Actinobacillus suis	407693457	---K-H-K-T-TD-T-----	YD	--E-IE-Q---LI---I-
	Actinobacillus ureae	322120930	---K-H-K-T-TD-T-----	YD	--E-IE-Q---LI---I-
	Avibacterium paragallinarum	523672791	-M-K-H-----AD-T-----	VQ	--Q-AE------IK----
	Pasteurella pneumotropica	517168114	---K-H--------------	VE	--Q-AE-R-D---K----
	Pasteurella bettyae	492139184	---K-H-----T--T-----	VE	-NQ-AE-M----I-----
	Actinobacillus succinogenes 13	152978358	-S-K-H--------T-----	VE	--Q-AE-A----I-----
	Mannheimia succiniciproducens	52424212	-S-K-H-----T--T-----	VE	--Q--E-L-D--I-----
	Aggregatibacter segnis	493771241	-V-K-H------D-T-----	VQ	--T-AE-P----IK----
	Aggregatibacter aphrophilus	491981671	---K-H------D-T-----	RE	--T-AE-P----IK----
	Aggregatibacter actinomycetemc	491778440	-V-K-H------D-T-----	HE	--T-AE-P----IK----
	Haemophilus somnus 2336	170718083	-R-K-H-----A--K-----	RE	--Q-AE------IL----
	Haemophilus somnus 129PT	113461773	-R-K-H-----A--K-----	RE	--Q-AE------IL--K-
	Haemophilus sp. oral taxon 851	497180830	---K-H--------K-----	VE	--Q--E-Q----IK----
	Haemophilus aegyptius	327473735	---K-H--------K-----	VE	--Q--E-Q-D--IK----
	Haemophilus parainfluenzae T3T	345429100	---K-H------D-K-----	VE	--Q--E------IK----
	Haemophilus haemolyticus	491864232	---K-H--------K-----	VE	--Q--E-Q-D--IK----
	Haemophilus influenzae 10810	378696770	---K-H--------K-----	VE	--Q--E-Q-D--IK----
	Haemophilus pittmaniae	494451577	-V-K-H-----AD-K-----	VE	--S-AE-------K----
	Haemophilus parasuis SH0165	219871676	---K-H-K-T--D-T-----	YD	--E-IE-Q---LI-A-V-
Other *Gammaproteo-bacteria* 0/250	Legionella nagasakiensis	209779033	-----E---R-I-------T	EK	T---A----S--I-----
	Marinobacter sp. ELB17	494591722	E----H---E-L--------	RE	--E----R-D--I-----
	Acinetobacter sp. NIPH 1847	491370379	--K-H-K-Q--D-K-----	YD	--E-IE-P----I-----
	Moritella sp. PE36	492899645	-----H---S-LD-S-----	VE	--E-AT-A--R-I-----
	Tatlockia micdadei	2627466	-V---E-----ID-Q----T	EK	T---AT--VS--I-----
	Thalassolituus oleivorans MIL-	473829413	-K-L-H-H-T-L--N-----	VK	--E-IA-G----I-----
	Shewanella sp. MR-4	113971139	-----H-H-T-ID-T-----	VE	--E-IA-P--R-I-----
	Psychromonas sp. CNPT3	470478469	---H-H-T-LD-T-----	VD	--E-I-------IK----
	Idiomarina loihiensis L2TR	56461409	-M-E-H-E-T-V--------	YE	--E-T--P--R-I-----
	Candidatus Legionella jeonii	4583155	-----D---T-LD-Q---TT	EK	T---AT--VS--I-----
	Oceanimonas sp. GK1	374334872	-V---H---T-VD-TQ----	VE	--E-A--P----I-----

Supplementary Figure 10

Partial sequence alignment for FKBP-type peptidyl-prolyl cis-trans isomerase. The protein contains 2 aa deletion that is specific for *Pasteurella sensu stricto.*

.

Group	Species	Accession	173		245
Pasteurella sensu stricto	Past. multocida subsp. m str. HN06	383311492	PASSILDIERKVTAKMRGDL		PIENFGAPLAGSLIPWIDK
	Past. multocida subsp. m str. Anand1_buffalo	401692294	--------------------		-------------------
	Past. multocida subsp. m str. Anand1_poultry	338218682	--------------------		-------------------
	Past. multocida subsp. m str. Anand1_poultry	338217898	--------------------		-------------------
	Past. multocida subsp. m str. 3480	386835511	--------------------		-------------------
	Past. multocida subsp. m str. HN06	380872764	--------------------		-------------------
	Past. multocida subsp. gallicida X73	404381296	--------------------		-------------------
	Past. multocida subsp. gallicida P1059	404381529	--------------------		-------------------
	Past. multocida subsp.m str.VTCCBAA264	662150000	--------------------		-------------------
	Past. multocida subsp. m str. PMTB	544579597	--------------------		-------------------
	Past. multocida subsp. m str. PMTB	557250343	--------------------		-------------------
	Past. multocida subsp. m str. HB03	570283089	--------------------		-------------------
	Past. multocida subsp. m str. Pm70	15603497	--------------------		-------------------
	Past. multocida subsp. m str. P52VAC	401688289	--------------------		-------------------
	Pasteurella multocida 671/ 90	512758580	--------------------		-------------------
	Pasteurella multocida 36950	356598033	--------------------		-------------------
	Pasteurella multocida 1500E	511370980	--------------------		-------------------
	Pasteurella multocida 93002	512754838	--------------------		-------------------
	Pasteurella multocida P1933	512755327	--------------------		-------------------
	Pasteurella multocida RIIF	512761301	--------------------		-------------------
	Pasteurella multocida 1500C	512764053	--------------------		-------------------
	Pasteurella dagmatis	260632372	----------R---------		--D----------------
Other Pasteurellales	Pasteurella bettyae	492142074	------E-------Q--SVE	F	-T-----A-G---------
	Mannheimia succiniciproducens	52424061	--------------E--A-S	F	-TD----A-----------
	Actinobacillus succinogenes 13	152977962	--------------E--A-S	F	-TD---TA-----------
	Aggregatibacter aphrophilus NJ	251793382	-H--------A------DSN	F	--D----------------
	Aggregatibacter actinomycetemc	491743607	-N--------A------DKD	F	--D----------------
	Aggregatibacter segnis	493771023	-N--------A------DNN	F	--D----------------
	Gallibacterium anatis UMN179	332289624	-S--------A------AED	F	-T-----A-G---------
	Actinobacillus suis H91-0380	407691599	-----------------E-S	F	-TD----------------
	Actinobacillus ureae	491830899	-----------------E-S	F	-TD----------------
	Actinobacillus pleuropneumonia	2494081	------E----------E-S	F	-TD----------------
	Actinobacillus capsulatus	517480854	-----------------E-G	F	-TD----------------
	Haemohilus ducreyi	33151282	--TC--A------DI--D-S	F	---H---------------
	Actinobacillus minor	492378846	------E----------AED	F	-TT----A-G---------
	Haemophilus aegyptius	327473112	--------------E--A-N	F	-TD----A-G---------
	Haemophilus somnus 2336	170718618	--T--------------AED	F	STT----A-----------
	Haemophilus somnus 129PT	113461688	--T--------------AED	F	STT----A-----------
	Haemophilus influenzae F3031	319897820	--------------E--S-S	F	-TD----A-G---------
	Haemophilus haemolyticus	491840026	--------------E--S-S	F	-TD----A-G---------
	Haemophilus parainfluenzae T3T	345429175	--------------E--S-S	F	-TD----A-G---------
	Haemophilus pittmaniae	494451354	--------------E--S-S	F	-TD----A-G---------
	Haemophilus sp. oral taxon 851	497184927	--------------E--S-S	F	-T-----A-G---------
	Haemophilus parahaemolyticus	491983155	--------------E--S-S	F	-T-----A-G---------
	Haemophilus paraphrohaemolytic	491991632	--------------A--S-S	F	-T-----A-G---------
	Haemophilus sputorum	494791543	--------------T--D-S	F	-TA----A-G---------
	Haemophilus parasuis SH0165	219871160	------E-------E--SED	F	-TA----------------
	Mannheimia haemolytica USDA-AR	472334224	-----------------AED	F	-T-----A-G---------
	Bibersteinia trehalosi USDA-AR	470167928	------E-------Q--A-D	F	-TA----A-G---------
	Pasteurella pneumotropica	517168125	--------------E--SAD	F	-T-----A-G---------
	Avibacterium paragallinarum	516415744	-----------------DAS	F	--D---T------------
Other Gammaproteo-bacteria 0/250	Dickeya dadantii Ech586	271502355	---A----------LS- GG	L	DV----V------------
	Dickeya zeae Ech1591	251787870	---A----------LS- GG	L	DV----V------------
	Plesiomonas shigelloides	499149527	---A-----------T-DGS	L	-VD---V------------
	Rahnella sp. Y9602	322830931	---A----------LT-SGT	L	-TD---V----G-------
	Hafnia alvei	490192463	---A---------ELG-SGT	L	-TD---V------------
	Yokenella regensburgei	493871072	---A---------SLT-SGE	L	AVD---V------------
	Enterobacter sp. Ag1	495733328	---A---------ELG-SGK	L	-VD---V------------
	Cronobacter malonaticus	495054021	---A----------LT-SGE	L	-VD---V----G-------
	Aeromonas diversa	491503773	---A-------I-E-T-SGT	L	-VD---V-----------T
	Pantoea sp. Sc1	496378765	---A------S--DFT-SGT	L	-TD---V------------
	Edwardsiella tarda EIB202	269140685	---A------R--DL--SGT	L	-TD---V------------
	Halomonas sp. TD01	497409267	TS-A----D-----A--SGD	F	-TD----------L----T
	Escherichia coli UTI89	91212916	-S-A---------TLT-SGE	L	-VD---V------------
	Yersinia enterocolitica subsp.	332163357	---A----------AT-SGK	L	-TD---V------------

Supplementary Figure 11

Partial sequence alignment for Aspartate-Semialdehyde Dehydrogenase. The protein contains 1 aa deletion that is specific for *Pasteurella sensu stricto.* No other species harbor this insert.

Group	Species	Accession	390	(insert)	427
Pasteurella sensu stricto	Pasteurella dagmatis	492154065	ALLGALVMVKLMLVGNDN	YD	SMVITIGKTFADLTGDKW
	Pasteurella multocida 671/90	512760234	----------------E-	--	------------V-----
	Pasteurella multocida 1500E	511370720	----------------E-	--	------------V-----
	Pasteurella multocida 1500C	512762880	----------------E-	--	------------V-----
	Pasteurella multocida 2000	512751484	----------------E-	--	------------V-----
	Pasteurella multocida 93002	512753070	----------------E-	--	------------V-----
	Pasteurella multocida P1933	512755610	----------------E-	--	------------V-----
	Pasteurella multocida RIIF	512761745	----------------E-	--	------------V-----
	Past. multocida subsp. m str. 3480	386835100	----------------E-	--	------------V-----
	Past. multocida subsp. gallicida P1059	404381029	----------------E-	--	------------V-----
	Past. multocida subsp. multocida P1062	557250787	----------------E-	--	------------V-----
	Past. multocida subsp. m str. Anand1_goat	338217416	----------------E-	--	------------V-----
	Past. multocida subsp. m str. Anand1_poultry	338217905	----------------E-	--	------------V-----
	Past. multocida subsp. m str. Pm70	15603717	----------------E-	--	------------V-----
	Past. multocida subsp. m str. Pm70	12722279	----------------E-	--	------------V-----
	Past. multocida subsp. m str. P52VAC	401687889	----------------E-	--	------------V-----
	Past. multocida subsp. m VTCCBAA264	662151847	----------------E-	--	------------V-----
	Past. multocida subsp. gallicida X73	404380743	----------------E-	--	------------V-----
	Past. multocida subsp. m str. HN06	383311110	----------------E-	--	------------V-----
Other *Pasteurellales*	Actinobacillus capsulatus	517482331	--I---I--N-------G		---KI---S--EA--EY-
	Pasteurella pneumotropica	517168237	--I---I--N-----G-A		---KI---S--QA--EY-
	Pasteurella bettyae	492137572	------I------I-GKE		---QI--QS--EY--QY-
	Mannheimia succiniciproducens	52424808	--I---I--N-----GEG		---K-------ET--EH-
	Actinobacillus succinogenes 13	152979623	--V---I--N-----G-G		---K-------ET--EH-
	Aggregatibacter segnis	493770012	--M------N-----GEH		---KI--R---EV---D-
	Aggregatibacter actinomycetemc	491712566	--V---I--N-----G--		---KI---S--EA--QY-
	Aggregatibacter aphrophilus NJ	251792350	--M------N-----GEH		---KI--R---EI--ND-
	Haemophilus somnus 2336	170717339	--V---I--N-----G-G		---KI---S--AA--EY-
	Haemophilus somnus 129PT	113460853	--V---I--N-----G-G		---KI---S--AA--EY-
	Gallibacterium anatis UMN179	332288638	--V---I--N-----G--		---KI---S--AA--EY-
	Actinobacillus suis H91-0380	407692534	--I---I--N-------G		---KI---S--EA--EY-
	Actinobacillus ureae	491832357	--I---I--N-------G		---KI---S--AA--EY-
	Actinobacillus pleuropneumonia	491783002	--V---I--N-------G		---KI---S--AA--EY-
	Actinobacillus minor	492374701	--I---I--N-----G-G		---KI---S--EA--EY-
	Bibersteinia trehalosi USDA-AR	470167286	-----II------I-GAD		---Q---RS--EF--QS-
	Mannheimia haemolytica USDA-AR	472334843	-----I-------I-GVD		---QI---N--EF--QS-
	Mannheimia haemolytica M42548	482885659	-----I-------I-GVD		---QI---N--EF--QS-
	Haemophilus parasuis SH0165	219870902	--V---I--N-----G-G		---KI--QS--QA--EY-
	Haemophilus sputorum	494787533	--I---I--N-----G-E		---KI--RS--HA--EY-
	Haemophilus parahaemolyticus	491987197	--I---I--N-----G-E		---KI--RS--HA--EY-
	Haemophilus paraphrohaemolytic	491993130	--I---I--N-----G-E		---KI--RS--HA--EY-
	Haemophilus pittmaniae	494450016	--T------N-----G-H		---KI--R---EI--ND-
	Haemophilus haemolyticus	491867161	--V------N-----GEH		---KI--R---EIS-SN-
	Haemophilus parainfluenzae	491966960	--M------N-----GEH		---KI--R---EV--SD-
	Haemophilus sp. oral taxon 458	545364985	--M------N-----GEH		---KI--R---EV---D-
	Haemophilus aegyptius	494054377	--V------N-----GEH		---KI--R---EIS-SN-
	Haemophilus influenzae R2866	309751139	--I------N-----GEH		---KI--R---EIS-SN-
Other *Gammaproteobacteria* 0/250	Cardiobacterium hominis	490242580	--F-------F-M--G--		---K---NS--QIA-TN-
	Photobacterium angustum	491509629	--V---I---F-MI-GEH		-PIM-T-NA----M-KN-
	Moritella sp. PE36	492900972	-I---I-----MA-GE-		-PILI--MA-S--M-TN-
	Vibrio scophthalmi	491781085	--V---I---F-MM-G-H		-PIM-T-RA-SE---TN-
	Yersinia ruckeri	490859449	---------NM-ML-GN-		AP--L---EL-SV--ES-
	Vibrio rotiferianus	498133176	--V---I---F-MM-DA-		-PIM-T-RALS--L-TS-
	Xenorhabdus nematophila ATCC 1	300722610	--F------NM-ML-DE-		-P-FI---AL-AM--EG-
	Serratia odorifera	491097500	------I--NM-MQ-G-R		AP--I--QAL--T--SG-
	Teredinibacter turnerae T7901	254787098	-------F-N--MM-GEQ		-A-AL--QSL-E---AY-
	Morganella morganii subsp. mor	455738355	--F---I--N--MQ-GE-		AP-YI--RSL-GA--ES-
	Alteromonas macleodii AltDE1	410862588	-------F-N--ML-GEQ		-A-SV---HM-Y-A-NN-

Supplementary Figure 12

A 2 aa insert in a conserved region of the lactate permease family transporter protein, specific *Pasteurella sensu stricto* is shown in this figure. No other species harbor this insertion.

```
                                                                      357                            387
Pasteurella   Pasteurella dagmatis ATCC 43325            260632997    VRIGNPLNITGVTHHV E NKPQYSTVLGLLQY
sensu stricto Pasteurella multocida 93002                512756477    --------------L- - --------------
              Pasteurella multocida 1500C                512763891    --------------L- - --------------
              Pasteurella multocida 1500E                511371845    --------------L- - --------------
              Pasteurella multocida 2000                 512750732    --------------L- - --------------
              Pasteurella multocida P1933                512756520    --------------L- - --------------
              Pasteurella multocida 671/ 90              512760522    --------------L- - --------------
              Pasteurella multocida RIIF                 512761903    --------------L- - --------------
              Pasteurella multocida 36950                378774785    --------------L- - --------------
              Past. multocida subsp. m str. Pm70         15602011     --------------L- - --------------
              Past. multocida subsp. m str. HN06         383310759    --------------L- - --------------
              Past. multocida subsp. m str. 3480         386834751    --------------L- - --------------
              Past. multocida subsp. m str. P1062        557251138    --------------L- - --------------
              Past. multocida subsp. m str.P52VAC        401690272    --------------L- - --------------
              Past. multocida subsp. m str. PMTB         544580907    --------------L- - --------------
              Past. multocida subsp. m  VTCCBAA264       662151314    --------------L- - --------------
              Past. multocida subsp. m str Anand1_goat   338219119    --------------L- - --------------
              Past. multocida subsp. m str Anand1_cattle 01701157     --------------L- - --------------
              Past. multocida subsp. m str Anand1_buffalo 401698941   --------------L- - --------------
              Past. multocida subsp. m str Anand1_poultry 338216942   --------------L- - --------------
              Past. multocida subsp. m str 3480          385201454    --------------L- - --------------
              Past. multocida subsp.gallicida X73        404383534    --------------L- - --------------
              Past. multocida subsp.gallicida P1059      404383534    --------------L- - --------------
              Past. multocida subsp. m P1062             557251138    --------------L- - --------------
              Past. multocida subsp. m str. HB03         570282365    --------------L- - --------------
Other         Haemophilus aegyptius                      327467404    ---AS------L-DY-   -R------V------
Pasteurellales Avibacterium paragallinarum               516416579    ----S-M----L-DY-   --------V------
              Pasteurella pneumotropica                  517168260    --V-S------L-DY-   -R------I------
              Haemophilus pittmaniae                     494451856    ----S------L-DY-   -R------V------
              Pasteurella bettyae                        492145430    --V-Y----S-L-DY-   -----A---------
              Mannheimia succiniciproducens              52425717     --V-Y------L-DY-   -----A---------
              Actinobacillus succinogenes 13             152979582    --V-S------L-DY-   -----A---------
              Aggregatibacter actinomycetemc             491735876    ----S------L-DY-   -----A--I------
              Aggregatibacter segnis                     493768770    ----S------L-DY-   --------I------
              Aggregatibacter aphrophilus NJ             251792027    --V-S------L-DY-   -----A--I------
              Gallibacterium anatis UMN179               332288548    -K--T------L-DY-   -----A---------
              Haemophilus influenzae 10810               378697366    ---AS------L-DY-   -R------V------
              Haemophilus pittmaniae                     494451856    ----S------L-DY-   -R------V------
              Haemophilus sp. oral taxon 851             497452636    ----S------L-DY-   -R------V------
              Haemophilus haemolyticus                   491875401    ----S------L-DY-   -R------V------
              Haemophilus parahaemolyticus               491987960    --V-S----A-L-DY-   -----A---------
              Haemophilus paraphrohaemolytic             491991888    --V------A-L-DY-   -----A---------
              Haemophilus parasuis SH0165                219870395    --V--------L-DY-   ---S-A---------
              Haemophilus sputorum                       494790961    --V------E-L-DY-   -----A---------
              Actinobacillus pleuropneumonia             126207510    --V-Y------L-DY-   -----A---------
              Actinobacillus ureae                       491831136    --V-Y------L-DY-   -----A---------
              Actinobacillus suis H91-0380               407693004    --V-Y------L-DY-   -----A---------
              Actinobacillus minor                       492358394    --V-Y------L-DY-   -----A-------F-
              Mannheimia haemolytica USDA-AR             472333002    --V-Y------L-DY-   -----A-A-------
              Bibersteinia trehalosi USDA-AR             470166543    --V-H------L-DY-   -----A---------
```

Supplementary Figure 13

A partial sequence alignment of the Cell division protein FtsA depicts the conserved 1 aa insertion shared by *Pasteurella sensu stricto.*

Group	Organism	GI	25		64
Aggregatibacter	Aggregatibacter actinomycetemcomitans D11S-1	261866907	FTGFGIGVGVGTTKYKDAK		RISNVDLIADYGIDYGNDFVG
	Aggregatibacter actinomycetemcomitans ANH9381	365966720	-------------------		---------------------
	Aggregatibacter actinomycetemcomitans D7S-1	387121099	-------------------		---------------------
	Aggregatibacter actinomycetemcomitans RhAA1	359757391	-------------------		---------------------
	Aggregatibacter actinomycetemcomitans SC1083	347992294	-------------------		---------------------
	Agg. actinomycetemcomitans serotype a str. A160	443549786	-------------------		---------------------
	Agg. actinomycetemcomitans serotype e str. SCC393	348003778	-------------------		---------------------
	Agg. actinomycetemcomitans serotype b str. S23A	443548871	-------------------		---------------------
	Agg. actinomycetemcomitans D11S-1	261412239	-------------------		---------------------
	Agg. actinomycetemcomitans serotype f str. D18P1	348001357	-------------------		---------------------
	Agg. actinomycetemcomitans serotype a str. H5P1	348002279	-------------------		---------------------
	Agg. actinomycetemcomitans serotype c str. SCC2302	348004682	-------------------		---------------------
	Agg. actinomycetemcomitans serotype b str. SCC1398	348006756	-------------------		---------------------
	Agg. actinomycetemcomitans serotype b str. I23C	348010515	-------------------		---------------------
	Agg. actinomycetemcomitans serotype c str. D17P-2	348656715	-------------------		---------------------
	Agg. actinomycetemcomitans ANH9381	365745633	-------------------		---------------------
	Agg. actinomycetemcomitans D7S-1	385875591	-------------------		---------------------
	Agg. actinomycetemcomitans Y4	429155976	-------------------		---------------------
	Agg. actinomycetemcomitans serotype c str. AAS4A	443542933	-------------------		---------------------
	Agg. actinomycetemcomitans serotype b str. SCC4092	443545908	-------------------		---------------------
	Agg. actinomycetemcomitans HK1651	601111157	-------------------		---------------------
	Aggregatibacter aphrophilus NJ	251793586	-----V--D----------		-----------------N---
	Aggregatibacter aphrophilus NJ8700	247534982	-----V--D----------		-----------------N---
	Aggregatibacter aphrophilus ATCC 33389	347814960	-----V--D----------		-----------------N---
	Aggregatibacter aphrophilus F0387	353345480	-----V--D----------		-----------------N---
	Aggregatibacter segnis	492001274	-----V---------E---		------------FNH-QNV--
Other *Pasteurellales*	Actinobacillus actinomycetemco	19911156	-------------------		---------------------
	Actinobacillus ureae	491831195	---V-V--DLT-V--DVDG	VKGK	QST-PA -VV---M----N-
	Actinobacillus pleuropneumonia	126207537	---V-V--DLT-V---VDG	VKGK	QSTGPA -VV---M---DN-
	Actinobacillus minor	492365015	---L-V--DLT-A---VDG	VKGK	QSTGPA -VV---M---SN---
	Actinobacillus suis H91-0380	407692976	---V-V--DLT-V--EVDG	VKGK	QSTGPA -VV---M----N-
	Haemophilus sputorum	494791298	---V-V--DLTSA---VDG	IKGK	QSTGPA -VV---M----NL--
	Haemophilus paraphrohaemolytic	491991617	---I-VSTDLT-A---ANG	VTGK	QSTGP- -VV---M----NL--
	Haemophilus parahaemolyticus	491987783	---V-VSADLT-A---VNG	VTGK	QSTGP- -VV---M----NL--
	Haemophilus parasuis SH0165	219871549	---G---MD-T-----NGD	LQGK	QA-GIN FTL--AT---RNLI-
	Haemophilus ducreyi 35000HP	33151516	---M---IDLD-A--TSHG	QNGK	RKA-PM IVL---M----NL--
	Mannheimia haemolytica USDA-AR	472335694	---I-V--D-T-----VEG	VKGK	DSTGPA -VV----EQ--N---
	Mannheimia haemolytica M42548	482885642	---I-V--D-T-----VEG	VKGK	DSTGPA -VV----EQ--N---
	Bibersteinia trehalosi USDA-AR	470167899	---V-V-LDLT-V---NSN	LQGK	QATGAN FVL--AL----NVL-
	Simonsiella muelleri	488718971	---ASV-TNIE-----IKN	SISG	ENTR-AV-KG-V-F-M--NL--

Supplementary Figure 14

A 4 aa deletion in a conserved region of the Outer membrane protein specific is for *Aggregatibacter*

			190		220
Aggregatibacter	Agg. actinomycetemcomitans ANH9381	365966332	TVILPTLARHHVNRD		DNVKSAVDFCNTMDWG
	Agg. actinomycetemcomitans D7S-1	387121526	---------------		----------------
	Agg. actinomycetemcomitans serotype b str. I23C	348008395	---------------		----------------
	Agg. actinomycetemcomitans serotype b str. S23A	443544851	---------------		----------------
	Agg. actinomycetemcomitans serotype b str. SCC1398	348006394	---------------		----------------
	Agg. actinomycetemcomitans serotype b str. A160	443550283	---------------		----------------
	Agg. actinomycetemcomitans Y4	429154918	---------------		----------------
	Agg. actinomycetemcomitans HK1651	601113024	---------------		----------------
	Agg. actinomycetemcomitans serotype b str. SCC4092	443547650	---------------		----------------
	Agg. actinomycetemcomitans serotype c str. SCC2302	348007426	---------------		----------------
	Agg. actinomycetemcomitans serotype c str. D17P-2	348656671	---------------		----------------
	Agg. actinomycetemcomitans serotype c str. AAS4A	443541805	---------------		----------------
	Agg. actinomycetemcomitans serotype d str. I63B	347996801	---------------		----------------
	Agg. actinomycetemcomitans serotype d str. H5P1	347997479	---------------		----------------
	Agg. actinomycetemcomitans serotype d str. D18P1	348002902	---------------		----------------
	Agg. actinomycetemcomitans serotype d str.SCC393	348003239	---------------		----------------
	Agg. actinomycetemcomitans serotype d str.D17P-3	348656236	---------------		----------------
	Agg. actinomycetemcomitans serotype d str. D7S-1	385876018	---------------		----------------
	Agg. actinomycetemcomitans D11S-1	261868574	----------Y----		----------------
	Aggregatibacter aphrophilus NJ	251794002	---------------		--A------R------
	Aggregatibacter aphrophilus ATCC 33389	347813475	---------------		--A------R------
	Aggregatibacter aphrophilus NJ8700	247535401	---------------		--A------R------
	Aggregatibacter aphrophilus F0387	353345600	---------------		--A------R------
	Aggregatibacter segnis	493770776	---------------		--A------R------
	Aggregatibacter segnis ATCC 33393	315476479	---------------		--A------R------
**Others** *Pasteurellales*	Haemophilus sp. oral taxon 851	497182424	--------------E	G	-SA------R------
	Haemophilus aegyptius	494053515	--------------E	G	-SA------R------
	Haemophilus influenzae 86-028N	68249552	--------------E	G	-SA------R------
	Haemophilus parainfluenzae T3T	345428924	--------------E	D	NSSQ-----RD-----
	Haemophilus haemolyticus	491853634	--------------E	G	-SD------R------
	Haemophilus pittmaniae	494452657	--------------T	D	-AEQ-GQ--HQ-----
	Avibacterium paragallinarum	516416005	--------------S	D	NAEQ-----R------
	Haemophilus somnus 129PT	113460561	-------S--Y---T	D	NIE------RQ-----
	Haemophilus somnus 2336	170718908	-------S--Y---T	D	NIE------RQ-----
	Pasteurella pneumotropica	517167578	--------------E	D	NSEQ-----R------
	Pasteurella dagmatis	492150466	------------S-H	D	NSAQ-E---R--L---
	Pasteurella multocida subsp. m	15603525	--------------H	D	HSSQ--I--RH-L---
	Pasteurella bettyae	492138635	-------S------S	D	-A---SE--RS-----
	Actinobacillus succinogenes	501021160	---------Q-ADCT	D	NAAQGVT--RQ-----
	Mannheimia succiniciproducens	52425810	-------S-Q----A	D	-VQ---A--RA-----
	Mannheimia haemolytica USDA-AR	472332644	--V--S-S-IAK-KE	L	TEIQRGEH-Q------
	Bibersteinia trehalosi USDA-AR	470166263	-----A-S-IAKQKT	L	SDEQRIQQ-KQ-----
	Actinobacillus capsulatus	517482124	--V--S-S-IAKKKE	I	-E-QR--E-QG-V---
	Actinobacillus pleuropneumonia	491797892	--V--S-S-IAKKKE	I	-E-QR--E-QG-----
	Actinobacillus ureae	491836958	--V--S-S-IAKKKE	I	-EAQR--E-QG-----
	Actinobacillus suis H91-0380	407693304	--V--S-S-IAKKKE	I	-DAQR--E-QG-----
	Actinobacillus minor	492353241	--V----S-IAKKK-	I	-DNQRK-E-QS-----
	Haemophilus parasuis SH0165	219871503	-----S-S-IAKQK-	L	TEEQRKFN-EQ-----
	Haemophilus ducreyi 35000HP	33152962	--V--N-S-ITKKKA	L	NEEQR-IA-KQ-L---
	Haemophilus paraphrohaemolytic	491988454	--V----SKIAKQKE	L	NETERKTQ-QT-----
	Haemophilus parahaemolyticus	491985107	--V----SKIAKQKA	L	NETARQAQ-QT-----
	Haemophilus sputorum	494790245	--V----SKIAKQK-	L	AETQRRE--Q--L---

Supplementary Figure 15

A 1 aa deletion in a conserved region of the Multidrug Transporter MurJ protein, specific *Aggregatibacter* is shown in the figure.

Group	Strain	ID	372	Insertion	412
Aggregatibacter	Agg. Actinomycetemcomitans D7S-1	387120244	GSLVSLSEFTAFGALSGKITGG	S	SMTIEGKIAQWVYVSLYR
	Agg. Actinomycetemcomitans HK1651	601112307	----------------------	-	------------------
	Agg. Actinomycetemcomitans D17P-3	348655454	----------------------	-	------------------
	Agg. Actinomycetemcomitans Y4	429152231	----------------------	-	------------------
	Agg. Actinomycetemcomitans RhAA1	359757161	----------------------	-	------------------
	Agg. Actinomycetemcomitans ANH9381	365967736	----------------------	-	------------------
	Agg. Actinomycetemcomitans D11S-1	261867944	----------------------	-	------------------
	Agg. Actinomycetemcomitans erotype e str. SCC393	387120244	----------------------	-	------------------
	Agg. Actinomycetemcomitans erotype e str. SCC1398	348006243	----------------------	-	------------------
	Agg. Actinomycetemcomitans erotype e str. AAS4A	443541389	----------------------	-	------------------
	Agg. Actinomycetemcomitans erotype e str. SCC4092	443547308	----------------------	-	------------------
	Agg. Actinomycetemcomitans erotype e str. SC1083	347993017	----------------------	-	------------------
	Agg. Actinomycetemcomitans erotype f str. D18P1	348000420	----------------------	-	------------------
	Agg. Actinomycetemcomitans erotype d str. I63B	347997013	----------------------	-	------------------
	Aggregatibacter segnis	493769445	---------------G------	-	-----------M------
Other *Pasteurellales*	Haemophilus haemolyticus	491870682	-T-----S---L-SI-N-FGKN		PL-VQ--F--FA------
	Haemophilus sp. oral taxon 851	497182862	-T-----S---L-SI-N-FGKN		PL-VQ--F--FA------
	Haemophilus parainfluenzae T3T	345429510	-T-----S---L-SI-N-FGKN		PL-VQ--F--FA------
	Haemophilus influenzae R2846	386266521	-T-----N---L-S-TN-FGKN		PL-VQ--F--FA------
	Haemophilus influenzae Rd KW20	16272688	-T-----N---L-S-TN-FGKN		PL-VQ--F--FA------
	Haemophilus influenzae 10810	378696947	-T-----N---L-S-TN-FGKN		PL-VQ--F--FA------
	Haemophilus influenzae F3031	319897753	-T-----N---L-S-TN-FGKN		PL-VQ--F--FA------
Other *Gammaproteobacteria* 0/250	Edwardsiella tarda EIB202	269139415	---------STV-S-M-NL-R-		--M---R--RIM------
	Citrobacter freundii	489928937	-------N-STV-S-M-NL-R-		--MV--R--RF--I----
	Aliivibrio salmonicida LFI1238	209694845	-------N-STV-S-M-NL-K-		--M---R--RI--I----
	Escherichia coli	485787518	-------N-STV-S-M-NL-R-		--M---R--RF--I----
	Shigella boydii	491148521	-------N-STV-S-M-NL-R-		--M---R--RF--I----
	Hahella chejuensis KCTC 2396	83649253	-------RYSTV-N-M-NL--K		--M---R--RF--I----
	Photobacterium damselae	491441018	-------R-STV-S-M-NL-K-		--MV--R--RV--I----
	Brenneria sp. EniD312	496403366	-------K-STV-S-M-NLMS-		-VM---R--RF--I----
	Salmonella enterica subsp. ent	197362727	-------N-STV-S-M-NL-R-		--M---R--RFL-I----
	Cronobacter sakazakii	495164111	-------K-STV-S-M-NLMR-		--M---R--RF--I----
	Serratia sp. M24T3	497321568	-------R-STV-S-M-NL-K-		--M---RL-RM-------
	Vibrio mimicus	491666772	-------R-STV-S-M-NL-R-		--MV--R--RV--I----
	Erythrobacter sp. NAP1	494285496	---I---R-STV-S-M-NLV--		A-AV--R--RFI-T----

Supplementary Figure 16

Partial sequence alignment of NADH dehydrogenase, showing the presence of 1 aa insertion that is specific for *Aggregatibacter*. The homologue of this is protein or the sequence of the region containing insertion is found to be missing in *Aggregatibacter aphrophilus*

```
                                                       418                                                    473
Mannheimia
Mannheimia haemolytica USDA-AR     472333011   TDDNPRTEDNQKIMADIVKGFSKP    QQIIHNREEAIKTAIEQAKATDVILIAGKGHE
M.haemolytica USDA-ARS-USMARC-185  472337351   ------------------------    --------------------------------
Mannheimia haemolytica D174        525663559   ------------------------    --------------------------------
Mannheimia haemolytica D171        523431845   ------------------------    --------------------------------
Mannheimia haemolytica PHL213      153094058   ------------------------    --------------------------------
Mannheimia haemolytica M42548      481063369   ------------------------    --------------------------------
Mannheimia haemolytica D193        528878678   ------------------------    --------------------------------
Mannheimia haemolytica USMARC_2286 526469516   ------------------------    --------------------------------
M. haemolytica USDA-ARS-USMARC-185 472261588   ------------------------    --------------------------------
Mannheimia haemolytica M42548      481063369   ------------------------    --------------------------------
Mannheimia haemolytica D153        523447566   ------------------------    --------------------------------
Mannheimia haemolytica D38         528825629   ------------------------    --------------------------------
Mannheimia haemolytica D35         528823205   ------------------------    --------------------------------
Mannheimia haemolytica MhBrain2012 528878382   ------------------------    --------------------------------
Mannheimia haemolytica MhSwine2000 528876521   ------------------------    --------------------------------
M. haemolytica ser. A2 str. OVINE  261308490   ------------------------    --------------------------------
M. haemolytica ser. A2 str. BOVINE 261310843   ------------------------    --------------------------------
M. haemolytica ser. 6 str. H23     452086134   ------------------------    --------------------------------
M.varigena USDA-ARS-USMARC-1312    575446106   ------------------------    ---------------A--T-Q-----------
M.varigena USDA-ARS-USMARC-1296    575444080   ------------------------    ---------------A--T-Q-----------
M.varigena USDA-ARS-USMARC-1388    575446833   ------------------------    ---------------A--T-Q-----------
M.haemolytica ser. A1/A6 str.PKL10 589591032   ---------------------ES- TA --------D-----------------------
M.varigena USDA-ARS-USMARC-1261    575440375   ---------------------ENQ SA --V----------------SI-----------
Other Pasteurellales
Mannheimia granulomatis            652755953   -----------N------------ QT V-------------------S-----------
Haemophilus aegyptius              494054439   -K------SQSQ-ET---A--KNM EK VG--PD-AQ--QF---S-VEN-----------
Haemophilus pittmaniae             494451972   -M-------PI--E---LA-VGDR NT VAL-T--QD--EF-VGG-IEE-----------
Pasteurella pneumotropica          517168269   -T-------PD--E----A---DM DK VGL-SD-AQ--EF--QS-QKN-L---------
Actinobacillus capsulatus          517480314   ----------T------L---VQV EK V-V--D--Q---S-----G-K-----------
Haemophilus influenzae             543952514   -K------SQNQ-E----A--KNM EK VG--PD-AQ--QF---S-VEN-----------
Haemophilus sp. oral taxon 851     497452628   -K------SQSQ-ES---A--KNM DK VG--PD--Q--QF---S-VEN-----------
Gallibacterium anatis              332288557   ---------AAA-IN---Q-LDF- NN AT---Q-KQ-LVY-FAC-N-N----V------
Haemophilus haemolyticus           491854538   -K------SQSQ-E----A--KNM DK VG--PD--Q--QF---S-VEN-----------
Avibacterium paragallinarum        523673909   ---------PAA--Q--QA--ENV DK VA---Q-DF--RY-LSL-QPR--V--------
Bibersteinia trehalosi USDA-AR     470166531   ---------PDV-F---MQ--H-- KA V-TM-I-QY--EQ-----SPK--V--------
Haemophilus parasuis SH0165        219870386   ---------PDV-FN-VM---R-- KA V-SM-I-QY--EQ--A--EPK-----------
Haemophilus paraphrohaemolytic     491991723   -----------------L---ENV GV V-V--D--Q------L---PQ-----------
Haemophilus parahaemolyticus       491987869   --------------V--L---ENV DV V-V--D--Q----T-L---PQ-----------
Haemophilus sputorum               494790980   -----------------L---ENV GI V-V--D--Q------L---PQ-----------
Haemophilus ducreyi 35000HP        33151486    -N-------QH--V-E-MQ--A-- DN ILV-YD--Q--QH--KH-SSA-L---------
Actinobacillus suis H91-0380       407693013   ----------T------L---VQV EK V-V--D--Q---------G-K-----------
Actinobacillus pleuropneumonia     491794283   ----------A------L---VQV EK V-V--D--Q---------NEK-----------
Actinobacillus ureae               491831120   ----------T----N-L---VQV EK V-V--D--Q---------NEK-----------
Actinobacillus minor               492350031   ---------PDV-FN--M---R-- KA V-SM-I-QY--EQ--A--EPK-----------
Actinobacillus succinogenes 13     152979591   ---------PE--V---TA--ARL KN V----A--A--CA--QS-AEN-----------
Mannheimia succiniciproducens      52425727    ---------RHQ-VQ---A--KR- ES VN-VYD--Q--R--VQS-VEN-----------
Pasteurella bettyae                492145494   ---------HH---Q---V--KQ- EQ IKV--D--Q----VQS-VEN-----------
Pasteurella dagmatis               492155823   --------AAE---Q--LL--EDQ SA VK-L-D--Q--AF-----DPE-T---------
Pasteurella multocida subsp. m     383310768   --------SAAQ-VQ--LA--EH- QH VEVC-A-DQ--IQ-LQK-DSD--V--------
Pasteurella multocida 36950        378774794   --------SAAQ-VQ--LA--EH- QH VEVC-A-DQ--IQ-LQK-DSD--V--------
Aggregatibacter segnis             493768749   ---------HTQ-----L---IH- QA V-V--Q-----A---QS-VEN-----------
Aggregatibacter aphrophilus NJ     251792018   ---------HTQ-----L---IN- QA V-V--Q-----A---KS-VEN-----------
Aggregatibacter actinomycetemc     491744776   -N----M-EPT------LT--VH- QR V-V--Q--Q--A---KS-VEN-----------
Haemophilus somnus 129PT           113460499   --------NPEQ--Q--LT--QQS SA V----Q--Q-----LQS-VEN--V--------
Haemophilus somnus 2336            170718778   --------NPEQ--Q--LT--QQS SA V----Q--Q-----LQS-VEN--V--------
Haemophilus parainfluenzae T3T     345429820   -Q-------PN--E---LT---RM ED VGV-PD------FT--N-VEN---V-------
Other Gammaproteobacteria 0/250
Thiomicrospira crunogena XCL-2     78484908    --------LPDA-V---K--MHRS -- VHVV-D-P---AF--KK-SSH-IVV-------
Cellvibrio japonicus Ueda107       192360758   -N----S--PAR-A----R-LNN- NG CLVLAD-AQ--DF-VQ---PG-LV--------
Francisella philomiragia           490415713   -E--N-F-SIES-FD--K----H- DK HTF-DS------YT--NS--N----L------
Vibrio mimicus                     491665840   ------S-SP-A-V--MLA-LTH- EL AH-E---FQ-ASY-LQN-G-Q-I--L------
Rahnella sp. Y9602                 322834425   ------S-EPRA-V---LA-LLDA GR V-E--G-A--VTS--M---ED--V--------
Aeromonas veronii                  491498935   --------EPAR----M-A-LRD- AA V-VE-D-VK--VL-TG--SKQ-I--V------
Pseudomonas sp. GM84               495372865   ---------P-G-F---RP--A-- AD VDFVAG-GQ--AQL-AS-A-D---VL------
Cronobacter universalis            494980781   ---------PKA-I---LT-MLDA GR ARVVEG-A--VTN--M--AEN--V-L------
Dickeya zeae Ech1591               251788245   ------S--P-A-VT--LS-LLDA GR V-V-AG-A--VTS-VM--A-D--V-V------
Xanthomonas campestris pv. cam     21230195    ------G--GDV-V---LR--AR- DA AIVQRD-AA--HQ--SM-G-D-IV--------
Hafnia alvei                       490196006   ------S-EPRS-----LN-MLDA GH A-A-SG-A--VTG--M---PD----V------
Vibrio sp. HENC-03                 497393525   ------S--PAL--K-MLA-LT-- AE AIVQ-D-FK-LSY-L-H-SSQ-I--L------
Salmonella enterica                486331335   --------EPRA-IN--LA-MLDA GQ VRVMEG-A--VTN--M---DN--V--------
```

Supplementary Figure 17

Partial sequence alignment for UDP-N-acetylmuramoylalanyl-D-glutamate--2,6-diaminopimelate ligase. The protein contains 2 aa deletion that is specific for *Mannheimia* is shown in the figure. *Mannheimia haemolytica ser. A1/A6 str.PKL10* and *Mannheimia varigena USDA-ARS-USMARC-1261* lacks this insert.

			504		531
Mannheimia	Mannheimia haemolytica USDA-AR	472333189	RTYDRQSAVDLVK	K	HHVDYHIRETFESA
	Mannheimia haemolytica D174	525663396	-------------	-	--------------
	Mannheimia haemolytica D171	523431632	-------------	-	--------------
	Mannheimia haemolytica D153	525702078	-------------	-	--------------
	Mannheimia haemolytica D35	528823164	-------------	-	--------------
	Mannheimia haemolytica USMARC_2286	526469350	-------------	-	--------------
	Mannheimia haemolytica D38	528826710	-------------	-	--------------
	Mannheimia haemolytica D193	528879592	-------------	-	--------------
	Mannheimia haemolytica USMARC_2286	526469350	-------------	-	--------------
	Mannheimia haemolytica M42548	482887395	-------------	-	--------------
	M. haemolytica USDA-ARS-USMARC-185	472261405	-------------	-	--------------
	M. haemolytica serotype 6 str. H23	452085964	-------------	-	--------------
	M. haemolytica M42548	481065996	-------------	-	--------------
	M. haemolytica serotype A1/ A6 str. PKL10	589590286	-------------	-	--------------
	M. haemolytica serotype A2 str. OVINE	261307780	-------------	-	--------------
	Mannheimia granulomatis	652757696	--------A----	-	-----YV-------
Other *Pasteurellales*	Bibersteinia trehalosi USDA-AR	470167741	-----R---K-L-		Q-I-FQV-------
	Haemophilus parasuis SH0165	219871186	-S---FNT-N-I-		-----QV-------
	Haemophilus ducreyi 35000HP	33151802	-----Y--IQ-T-		L---FQ--------
	Haemophilus sputorum	494789674	------TT-G-I-		QD-NFIV-------
	Haemophilus parahaemolyticus	491988280	------TT-S-I-		QD--FIV-------
	Haemophilus paraphrohaemolytic	491992094	------TT-S-I-		QD--FIV-------
	Actinobacillus suis H91-0380	407691804	-----H--IE-T-		R---FQ--------
	Actinobacillus ureae	491835981	-----H--IE-T-		L---FQT-------
	Actinobacillus pleuropneumonia	491785098	-----H--IE-T-		L---FQ----L---
	Haemophilus pittmaniae	494450521	-----RT--E-I-		YD--FIV-------
	Haemophilus parainfluenzae T3T	345429902	-----RTT-N-I-		QD--FIV-------
	Haemophilus sp. oral taxon 851	497182364	-----KTT-S-I-		QD--FIV-------
	Haemophilus influenzae 86-028N	68249497	-----KTT-S-I-		QD--FIV-------
Other *Gammaproteo-bacteria* 0/250	Acinetobacter junii	491093379	-S---EHSLH---		-N---M--------
	Acinetobacter ursingii	491131668	-S---EHSLS---		KD---Q--------
	Xanthomonas sacchari	498028753	-S---EH-LE-IA		AG------------
	Acinetobacter sp. CIP 70.18	491344899	-S---EHSLY---		-K---M--------
	Pseudomonas mendocina	489337704	-AF--EH-LE---		-DA--IV-------
	Pseudomonas stutzeri ATCC 1758	339494315	-AF--EH-LE---		-DA--IV-------
	Pseudoxanthomonas spadix BD-a5	357417720	-SF--EH-LQ---		AG---Q--------
	Xanthomonas axonopodis pv. cit	21243026	-S----HSLQ--A		AG---Q--------
	Xanthomonas campestris pv. ves	78048045	-S----HSLQ--A		AG---Q--------
	Pseudomonas putida KT2440	26990029	-AF--EH-LE---		-DA--IV------S
	Serratia plymuthica	493362897	-AH--EH-IE-I-		AN---QV-V-----
	Alcanivorax pacificus	496012633	-A---RH-LT-IR		DD--FLV-------

Supplementary Figure 18

Partial sequence alignment for Glutathione-regulated potassium-efflux system protein is shown in figure. The protein contains 1 aa insertion that is specific for *Mannheimia*.

Group	Organism	ID	214		252
Mannheimia	Mannheimia haemolytica USDA-AR	472334521	FNKILNSEVIQAAIAEEAKKS		SPEKARKEAEKIINEIAA
	Mannheimia haemolytica D35	528823843	---------------------		------------------
	Mannheimia haemolytica D193	528871831	---------------------		------------------
	Mannheimia haemolytica M42548	482885978	---------------------		------------------
	Mannheimia haemolytica D174	525664688	---------------------		------------------
	Mannheimia haemolytica D171	525657694	---------------------		------------------
	Mannheimia haemolytica D153	525703384	---------------------		------------------
	Mannheimia haemolytica USMARC_2286	526468127	---------------------		------------------
	Mannheimia haemolytica PHL213	153094325	---------------------		------------------
	Mannheimia haemolytica D38	528824437	---------------------		------------------
	M. haemolytica Ser. A1/ A6 str. PKL10	589590824	---------------------		------------------
	M. haemolytica ser. A2 str. BOVINE	261310643	---------------------		------------------
	M. haemolytica ser. 6 str. H23	452086460	---------------------		------------------
	M. haemolytica USDA-ARS-USMARC-183	472258758	---------------------		------------------
	M. haemolytica USDA-ARS-USMARC-185	472260218	---------------------		------------------
	M. haemolytica MhSwine2000	528873636	---------------------		------------------
	M. haemolytica MhBrain2012	528877526	---------------------		------------------
	M. varigena USDA-ARS-USMARC-183	472334521	---------------------		------------------
	M. varigena USDA-ARS-USMARC-185	472335981	---------------------		------------------
	M. varigena USDA-ARS-USMARC-1261	575441602	---------------------		------------------
	M. varigena USDA-ARS-USMARC-1312	575445157	---------------------		------------------
	M. varigena USDA-ARS-USMARC-1388	575447863	---------------------		------------------
	M. varigena USDA-ARS-USMARC-1296	575443019	---------------------		-L-----------H----
	Mannheimia granulomatis	652756314	---------------------		------------------
Other *Pasteurellales*	Haemophilus ducreyi	33151743	-D-LIQLPT-VQ--ED---TK	KI	PIP---Q-----LD----
	Avibacterium paragallinarum	516417625	---L-QLPT-LK--ED---SK	KI	PL---H---Y--LD----
	Actinobacillus capsulatus	517482099	L--LIQLPT-VQ--ED---SK	KI	-K---QQ-----LD----
	Pasteurella pneumotropica	517166617	---L-Q-AP-----DD---SK	NI	-R---Q---Y--LD----
	Bibersteinia trehalosi USDA-AR	470167473	---L-Q-PA-L---ED----P	KS	------------MD----
	Haemophilus parasuis SH0165	219870296	---L-Q-PA-L---ED----P	KS	-L----------LD----
	Haemophilus sputorum	494789007	---L-D-AALKK---D--ENK	KI	------Q-----LE----
	Haemophilus parahaemolyticus	491986948	---L-D---LKK---D--ENK	KI	PL----A--T-MLD----
	Haemophilus paraphrohaemolytic	491989884	---L-D-D-LKK---D--ENK	KI	PL----A--A-MLD----
	Actinobacillus minor	492357411	---L-D---LKK---D--ESK	KI	------Q-----L-----
	Actinobacillus pleuropneumonia	190150433	----IQ-PA-K---E----TK	KI	-I----Q-----V-----
	Actinobacillus ureae	491831417	---LIQLSA-VQ--ED---SK	KI	-K---QQ-----LD----
	Actinobacillus suis H91-0380	407692764	---LIQLPA-VQ--ED---SK	KI	-K---QQ-----LD----
	Actinobacillus succinogenes 13	152979421	---L-QHPAVLQ--ED--RGK	NM	TK----------L-----
	Mannheimia succiniciproducens	52306852	---L-Q-P--LS---D---SK	NM	-R-R-HQ-----LK----
	Pasteurella bettyae	492142581	---L-Q-P--LE---D---SK	NM	-QAR--Q-----LK----
	Pasteurella dagmatis	492155126	---L-V-SA-LD--KD---AK	NI	-E---TQ--K--LE----
	Pasteurella multocida subsp. m	383311863	---L-V-PT-LD--QD---AK	NI	-E---TQ--K--LD----
	Gallibacterium anatis UMN179	332288840	-A-L--N-K-IK---D--ENK	KI	------Q-----LQ----
	Aggregatibacter actinomycetemc	365966896	---L-QQP--L---ED---SK	SI	-V----------LD----
	Aggregatibacter segnis	493768272	---L-QLP--L---ED---SK	NI	-R---H------LD----
	Haemophilus somnus 129PT	113460531	---L-Q-PA-LN---D---SK	RI	-K-------Y--LD----
	Haemophilus aegyptius	494053302	---L-Q--A-RR--ED---SK	NI	-I---Q---Y--LD----
	Haemophilus influenzae 10810	378696948	---L-Q--A-RR--ED---SK	NI	-I---Q---Y--LD----
	Haemophilus influenzae F3047	319775311	---L-Q--A-RR--ED---SK	NI	-I---Q---Y--LD----
Other *Gammaproteobacteria* 0/250	Morganella morganii subsp. mor	455737880	---L-K-KA-EK-VED---AK	KI	TI----QN-VSMM--V--
	Xenorhabdus nematophila	498913621	-K-L-A-KA-EK---D-VRSK	KI	--D--KQN-INMLE----
	Brenneria sp. EniD312	496405590	---L---KA-KK--DD--RSK	KL	-H-R-QQN-IALME----
	Rahnella sp. Y9602	322834784	---L-A-KA-EK--ED--RSK	KI	-H---QQN-IALME----
	Sodalis glossinidius str. 'mor	85060120	---L-A-KA-EK-VED--RSK	KI	-V---QQN-IELME----
	Alteromonas macleodii str. 'Bl	407701965	-SV-G-DAVRR-----SRSK	KV	-H---KET-QSY-T----
	Hafnia alvei	490196787	---L-S-KA-EK-VED--RSK	KI	-R---QQN-VALME----
	Yersinia enterocolitica subsp.	386310208	-K-L-T-KA-EK-V-D--RTK	KI	-H---QQN-ITLME----
	Vibrio parahaemolyticus	491619082	-KRLMK--A-EQ--LD--NSK	DI	PI-------HA-MD----
	Escherichia coli	485767479	---L-A-RA-AK-VED--RSK	KI	-H---QQN-IALME----
	Salmonella enterica	486386721	---L-A-KA-AR-VED--RSK	KI	-H---QQN-IALME----
	Shimwellia blattae DSM 4481 =	387890936	---L-A-KA-AK-VED--RSK	KI	-H---QQN-VALME----

Supplementary Figure 19

A 2 aa deletion in a partial sequence alignment of the Glycerol-3-phosphate acyltransferase protein specific for *Mannheimia* is depicted in the figure.
